# Novel lectin-based chimeric antigen receptors target Gb3-positive tumour cells

**DOI:** 10.1007/s00018-022-04524-7

**Published:** 2022-09-12

**Authors:** Ana Valeria Meléndez, Rubí M.-H. Velasco Cárdenas, Simon Lagies, Juliane Strietz, Lina Siukstaite, Oliver S. Thomas, Jana Tomisch, Wilfried Weber, Bernd Kammerer, Winfried Römer, Susana Minguet

**Affiliations:** 1 Faculty of Biology, University of Freiburg, Schänzlestraße 1, 79104 Freiburg, Germany; 2 BIOSS, Centre for Biological Signalling Studies, University of Freiburg, Schänzlestraße 18, 79104 Freiburg, Germany; 3 CIBSS, Centre for Integrative Biological Signalling Studies, University of Freiburg, Schänzlestraße 18, 79104 Freiburg, Germany; 4 Spemann Graduate School of Biology and Medicine (SGBM), University of Freiburg, Albertstraße 19a, 79104 Freiburg, Germany; 5 Institute of Organic Chemistry, Albert-Ludwigs-University Freiburg, Albertstraße 21, 79102 Freiburg, Germany; 6 CYTENA GmbH, Zollhallenstr. 5, 79106 Freiburg, Germany; 7 Centre for Integrative Signalling Analysis, University of Freiburg, Habsburgerstraße 49, 79104 Freiburg, Germany; 8 Freiburg Institute for Advanced Studies (FRIAS), University of Freiburg, Freiburg, Germany; 9 Center of Chronic Immunodeficiency (CCI), University Clinics and Medical Faculty, Freiburg, Germany

**Keywords:** Lectin, CAR T cells, Cancer, Cell death, Tumour-associated carbohydrate antigens, Glycosphingolipid targeting, Globotriaosylceramide, Killing, Glycans, Gb3, Carbohydrates Immunotherapy Aberrant glycans

## Abstract

The link between cancer and aberrant glycosylation has recently become evident. Glycans and their altered forms, known as tumour-associated carbohydrate antigens (TACAs), are diverse, complex and difficult to target therapeutically. Lectins are naturally occurring glycan-binding proteins  that offer a unique opportunity to recognise TACAs. T cells expressing chimeric antigen receptors (CARs) have proven to be a successful immunotherapy against leukaemias, but so far have shown limited success in solid tumours. We developed a panel of lectin-CARs that recognise the glycosphingolipid globotriaosylceramide (Gb3), which is overexpressed in various cancers, such as Burkitt's lymphoma, colorectal, breast and pancreatic. We have selected the following lectins: Shiga toxin's B-subunit from *Shigella dysenteriae*, LecA from *Pseudomonas aeruginosa*, and the engineered lectin Mitsuba from *Mytilus galloprovincialis* as antigen-binding domains and fused them to a well-known second-generation CAR. The Gb3-binding lectin-CARs have demonstrated target-specific cytotoxicity against Burkitt's lymphoma-derived cell lines as well as solid tumour cells from colorectal and triple-negative breast cancer. Our findings reveal the big potential of lectin-based CARs as therapeutical applications to target Gb3 and other TACAs expressed in haematological malignancies and solid tumours.

**Supplementary Information:**

The online version contains supplementary material available at 10.1007/s00018-022-04524-7.

## Introduction

The successful treatment of B-cell malignancies with chimeric antigen receptor (CAR) T cells has demonstrated the therapeutical potential of cell-based immunotherapies against cancer [1, 2]. CARs are engineered receptors that redirect and activate T cells upon encountering a tumour-specific antigen to eventually eliminate cancer cells. CAR T cells combine an antigen-binding domain, traditionally a single-chain variable fragment (scFv), with a transmembrane domain and an intracellular signalling domain [3]. To date, CAR T cells primarily use scFv derived from antibodies targeting cell surface proteins, such as CD19 on B lymphocytes. However, one of the main mechanisms to overcome CAR recognition of cell surface proteins is antigen escape, which leads to recurrence after treatment [4, 5].

  In humans, cell membrane is often decorated with surface proteins and lipids which are known or predicted to be glycosylated. Cell surface glycans participate in critical cellular processes such as cell–cell communication, signalling, host-pathogen interactions and disease development and progression. Given the importance of glycosylation, it is not surprising that minor changes in this process profoundly impact cell biology [6, 7]. The dysregulation of the glycan biosynthetic pathway is reflected as aberrant glycosylation and leads to malignant transformation [8, 9]. Altered glycan profiles include the appearance of truncated or novel structures, accumulation of precursors, and loss or overexpression of specific glycan structures on cancer cell surfaces named tumour-associated carbohydrate antigens (TACAs) [10, 11]. Therefore, targeting glycans could be a broader and more effective anti-cancer strategy than targeting subjacent core proteins [12]. Glycan-binding proteins, namely antibodies and lectins, which recognise altered glycan profiles in malignant cells, might provide an instrumental basis for tumour-targeted immunotherapy [13, 14]. On the one hand, carbohydrate-binding antibodies have been investigated for several decades to target TACAs. However, only a few were approved for clinical use due to reduced antibody recognition as a consequence of the wide variety of glycan epitopes [11, 15, 16]. On the other hand, lectins are proteins that recognise and bind carbohydrate moieties present in lipids and proteins, being valuable tools in biotechnology and medicine. Lectins have been used for cancer diagnosis, imaging [17, 18], and are currently explored as drug delivery systems [19–21]. Thus, lectins represent a promising addition to antibody-based applications. Most TACAs, particularly glycosphingolipids (GSLs) are poor immunogens [22], and it is not easy to generate high-affinity antibodies that could be applied in immunotherapy. For this reason, novel non-antibody-based approaches should be developed to target TACAs.

Most human cancers exhibit alterations in GSLs composition and metabolism, including those from the globo-series. The association of GSLs with cancer was first reported more than 50 years ago [23]. Among the globo-series, the globotriaosylceramide (Gb3, also known as CD77 or P^k^ antigen) [24, 25] was first identified as a fibrosarcoma-associated antigen [26]. Gb3 is present in the extracellular leaflet of the plasma membrane [27, 28]. It is composed of a ceramide backbone linked to a neutral trisaccharide made of galactose (Gal) and glucose (Glc) (Galα1-4Galβ1-4Glc) [24, 29]. The ceramide consists of sphingosine and a fatty acyl chain with different lengths, degrees of saturation and hydroxylation [29–31], determining how single Gb3 molecules are embedded in the plasma membrane and the presentation of the trisaccharide head group of Gb3. Gb3 is highly expressed in Burkitt's lymphoma [32], colorectal carcinoma [33], breast [34], ovary, and pancreatic cancer [35], as well as gliomas [36] and acute non-lymphocytic leukaemia (ANLL) [37]. Furthermore, Gb3 has been correlated with invasiveness, metastasis, angiogenesis and multi-drug resistance [38–42]. Overall, Gb3 might be an attractive target for engineered CAR T cells. Indeed, numerous lectins from unique origins have specificity for Gb3 [43], such as LecA from *Pseudomonas aeruginosa* [44], the non-toxic B-subunit of Shiga toxin (StxB), produced by *Shigella dysenteriae* and enterohemorrhagic strains of *Escherichia coli* (EHEC) [45], and the computationally redesigned Mitsuba [46] from the shellfish lectin MytiLec-1 [47].

In the present study, we successfully generated a panel of CARs, each of them with a Gb3-binding lectin as antigen-binding domain linked to the intracellular signalling domains from 41BB and CD3ζ. Lectins with different molecular weights and architectures but a common specificity towards Gb3 were chosen. In vitro analyses revealed that lectin-CAR T cells specifically recognised Gb3^+^ cancer cells and were cytotoxically active against cell lines from Burkitt's lymphoma, colorectal and breast cancer.

## Material and methods

### Cell lines

Ramos, Raji and Namalwa cells were grown in Roswell Park Memorial Institute (RPMI) 1640 medium. HT-29, HCT-116, LS-174 cells were cultured in Dulbecco's Modified Eagle Medium (DMEM) with 4.5 g L^−1^ glucose, l-glutamine and non-essential aminoacids (NEAA). MDA-MB-231 and JIMT1 cells were grown in DMEM with 1 g L^−1^ glucose. MDCK Gb3^+^ and MDCK WT were cultured in DMEM with 4.5 g L^−1^ glucose, L-glutamine. Media were supplemented either with 5% (for MDCK cell lines) or 10% (other cell lines) fetal calf serum (FCS) and antibiotics; the cells were cultured at 37 °C and 5% CO_2_ MDCK cells expressing Gb3 were established by stable overexpression of Gb3 synthase as described earlier [21, 44]. Daudi cells were grown in Iscove's Modified Dulbecco's Medium (IMDM) and HEK293T cells in DMEM, both media supplemented with 10% fetal bovine serum and antibiotics; cells were cultured at 37 °C and 7.5% CO_2_.

### Antibodies

The following antibodies were applied for activation: anti-CD28.2 (BioLegend #302902), anti-mouse CD3ε (145-2C11, here named 2C11, from J. Bluestone, San Francisco, CA, USA). An anti-ζ antiserum 449 and a secondary antibody anti-mouse horseradish peroxidase (HRP)-conjugated (Cell Signaling #7076S) were used for immunoblotting. The following antibodies were utilised for flow cytometry: APC-labeled anti-FLAG (#637307), Alexa Fluor 488-labelled anti-CD3 (#300415), PE-labeled anti-hCD25 (#302606) all from BioLegend. APC-labelled anti-hCD69 (#MHCD6905) from Invitrogen, APC-labeled anti-hCD8 (#IM2469) from Beckman Coulter and PE-Cy7-labeled anti-CD137 (#25-1371-82) from eBioscience.

### Generation of CAR constructs

To generate CARs targeting Gb3, the sequences encoding the B-subunit of Shiga toxin (StxB) from *S. dysenteriae*, LecA from *P. aeruginosa,* and Mitsuba, a variant of the shellfish lectin MytiLec-1, were cloned in frame with the lentiviral vector pCDH-EF1-19BBζ-T2A-copGFP. The lectin amino acid sequences were obtained from Uniprot or PDB, and the synthesised gene from Integrated DNA Technologies. The lentiviral vector was a gift from TCR2 Therapeutics. This second-generation CAR consists of the scFv from murine anti-hCD19 (FMC63) fused to the extracellular and transmembrane region of human CD8α, the 41BB endodomain and the ζ cytoplasmic tail signalling domain under the control of an EF-1α promoter. The sequence of each cloned CAR was verified by Sanger sequencing.

### Lentivirus production and lentiviral transduction

A total of 10^7^ HEK293T cells were plated and incubated overnight. After the medium exchange, the HEK293T cells were transfected with the packaging plasmid pMD2.G, the gag/pol pCMVR8.74 and the respective construct using PEI (Polysciences) transfection. The medium containing lentiviral particles was collected at 24 and 48 h post-transfection and concentrated by a 10% sucrose gradient. After 4 h of centrifugation at 10,000 rpm and 6 °C, the supernatant was discarded, and the virus pellet was resuspended in phosphate-buffered saline (PBS) to be stored at − 80 °C.

### Activation of primary human T cells, transduction and expansion

Peripheral blood mononuclear cells (PBMCs) were isolated from fresh blood of healthy donors using density centrifugation (Pancoll). PBMCs were resuspended in RPMI medium with 1000 U mL^−1^ of recombinant IL-2 (Prepotech) and activated with anti-CD3/CD28 (1 mg mL^−1^) antibodies. After 48–72 h, the remaining cells, mostly T cells, were lentivirally transduced using spin transfection (1800 rpm for 90 min and 30 °C) in the presence of 5 mg mL^−1^ protamine sulfate (Sigma) and 1000 U mL^−1^ IL-2 with a multiplicity of infection (MOI) of 4. Cells were maintained in a medium supplemented with 100 U mL^−1^ IL-2 for a maximum of 6–7 days after transduction and before using them for the indicated experiments. Activated but non-lentiviral transduced cells (NT) were included as a control.

### CAR expression

Transduction efficiency was monitored by BFP expression after 4–6 days. The CAR surface expression was verified with an anti-FLAG antibody by flow cytometry. Samples were analysed using a GALLIOS flow cytometer (Beckman Coulter), and data analysis was performed using FlowJo V.10. The presence of the CARs after transduction was also confirmed by immunoblotting. A total of 5 × 10^5^ transduced cells were resuspended in 20 µL of 200 mM Tris. The samples were split into two equal parts, and cells were lysed either with reducing sodium dodecyl sulfate (SDS) buffer or non-reducing buffer plus 5 µL of urea. The samples were boiled for 5 min at 95 °C. Then the proteins were separated on 8% Tris–glycine gels and transferred onto a nitrocellulose membrane. Primary antibody anti-ζ chain (1:1000) was added and incubated overnight at 4 °C, and the corresponding-conjugated secondary antibody (1:10,000) was incubated with the membrane for an hour at room temperature (RT). Finally, ClarityTM Western ECL Chemiluminescent Substrate (Bio-Rad) was used to detect the luminescence signal by a Vilber Lourmat Fusion FX chemiluminescence imager.

### Cytotoxicity assay

For the bioluminescence-based cytotoxicity assay, luciferase-expressing tumour cells (Ramos, Daudi, Raji, Namalwa, HT-29, HCT-116, LS-174, MDA-MB-231, JIMT1, MDCK Gb3^+^ or MDCK WT, as indicated) were plated at a concentration of 5 × 10^4^ cells mL^−1^ (adherent cells, day before) or 1 × 10^5^ cells mL^−1^ (suspension cells, day of the assay) in 96-well flat-bottom plates in triplicates. Then, 75 μg mL^−1^ of D-firefly luciferin potassium salt (Biosynth) were added to the tumour cells, and bioluminescence (BLI) was measured in the luminometer (Tecan infinity M200 Pro) to establish the BLI baseline. Subsequently, CAR T cells were added in a 5:1 effector to the target (E:T) cells ratio and incubated for 12, 17, 24 or 48 h (as indicated) at 37 °C. BLI was measured as relative light units (RLUs). RLU signals from cells treated with 1% Triton X-100 indicate maximal cell death. RLU signals from tumour cells incubated with non-transduced T cells determine baseline cell death. Percent specific lysis (specific killing) was calculated with the following formula: % specific lysis = 100 × (average baseline death RLU − test RLU) / (average baseline death RLU − average maximal death RLU).

For the live imaging cytotoxicity assay, GFP-expressing tumour cells (HT-29 and LS-174) were plated at a concentration of 10^4^ cells per well one day before in a 96-well flat microtiter plate (Corning, #3595) in quadruplicates. When indicated, cells were stained with the C.LIVE Tox Red dye (CYTENA, #12144) to a final concentration of 0.5 µM on the day of the experiment. Then, CAR T cells were added in a 5:1 E:T cells ratio and monitored every 3 h for 48 h using the live cell imaging system CELLCYTE X (CYTENA). The total area of GFP and C.LIVE Tox Red fluorescence was quantified using the analysis feature in CELLCYTE Studio.

### Cytokine detection

Cytokine release was tested using a commercially available enzyme-linked immunoabsorbent assay (ELISA) kit for tumour necrosis factor α (TNF-α) and interferon γ (IFN-γ) (Invitrogen). ELISAs were conducted according to the manufacturer's instructions. All CAR T cells were co-cultured with Ramos or Namalwa cells to a 5:1 effector to target (E:T) ratio for 48 h in triplicate wells. The supernatant was collected, and the TNF-α and IFN-γ concentrations were measured in a microplate reader (Synergy H4, Biotek Instruments).

### Activation of CAR T cells

Activation of CAR T cells was assessed by co-culturing CAR T cells with target cells for 48 h to a 5:1 E:T ratio in triplicate wells**.** T cells were stained with anti-CD3, anti-CD25, anti-CD8 and anti-CD137 antibodies. Samples were analysed using the Attune NxT flow cytometer (Thermo Fisher), and data analysis was performed using FlowJo V.10.

### Gb3 inhibition by PPMP treatment

Ramos cells, 2 × 10^5^ cells per well, were seeded into a six-well plate and exposed to 1 and 2 μM of 1-phenyl-2-palmitoylamino-3-morpholino-1-propanol (PPMP from Enzo Life Sciences) for 48 h. At this time point, cells were washed with PBS, and fresh PPMP was added. 24 h later, Gb3 was detected by staining the cells with labelled StxB (Sigma Aldrich) and analysed using the GALLIOS flow cytometer. The data were analysed using FLowJo V.10. To verify cell viability after PPMP treatment, we used a cell proliferation and viability kit (Millipore Sigma) and followed the manufacturer's instructions. 1 × 10^4^ Ramos cells were seeded and supplemented with 10 µL of the MTT labelling reagent. After 4 h, 100 µL of solubilisation solution were added and incubated overnight at 37 °C, 5% CO_2_. Cell viability was measured at the microplate reader (Synergy HT, Biotek Instruments).

### Gb3 profiling by LC/MS analysis

One day before the analysis, Ramos, Namalwa, HT-29, LS-174 and MDA-MB-231 cells were seeded in six-well plates. Gb3 composition analysis was done as described previously [48]. In brief, cells were washed with 0.9% NaCl and quenched with 1 mL methanol:water (1:1, v/v). Then, the cells  were detached or collected and transferred to a new tube and lysed with 500 µL of a chloroform solution with 2 µg mL^−1^ heptadecanoic acid by vigorous vortexing. 200 µL of the lower phase were evaporated and analysed by targeted LC–MS analysis. Chromatographic separation was achieved on a BEH-C18 column (waters, 2.1 × 100 mm, 1.8 µm). It was used with A, a gradient acetonitrile:H_2_O 6:4, 10 mM ammonium formate, 0.1% formic acid, and B, isopropanol:acetonitrile 9:1, 10 mM ammonium formate, 0.1% formic acid [49]. A triple quadrupole mass spectrometer (Agilent Technologies, G6460A) was operated in dynamic multiple reaction monitoring. Three experimental replicates were analysed in one LC–MS sequence. Samples were injected randomly, and a pooled quality control sample was regularly injected. Gb3 species were normalised to the internal standard and peak sum of detected sphingolipids. MetaboAnalyst 5.0 [50] was used for visualisation. Data were range-scaled for the heat map.

### Composition and preparation of giant unilamellar vesicles

Giant unilamellar vesicles (GUVs) were composed of 1,2-dioleoyl-sn-glycero-3-phosphocholine (DOPC), cholesterol (both Avanti Polar Lipids) Atto 647N 1,2-dioleoyl-sn-glycero-3-phosphoethanolamine (DOPE, Sigma Aldrich), and Gb3 (Matreya) at a molar ratio of 59.7: 30: 0.3: 10 mol%, respectively. GUVs were prepared by the electroformation method described by Madl et al. 2016. Shortly, lipids dissolved in chloroform with a total concentration of 0.5 mg mL^−1^ were spread on indium tin oxid-covered (ITO) glass slides and dried in a vacuum for at least one hour or overnight. Two ITO slides were assembled to make a chamber filled with sucrose solution adapted to the osmolarity of the imaging buffer Hanks' Balanced Salt Solution (HBSS, Thermo Fisher Scientific). Then, an alternating electrical field with a strength of 1 V mm^−1^ was applied for 2.5 h at RT. Later, we observed the GUVs in chambers manually built as described elsewhere [51].

### Live-cell imaging

On the day of the experiment, BFP-sorted cells were stained with a green CellTrace™CFSE proliferation kit (Thermo Fisher Scientific) following the manufacturer's instructions and rested in standard culture conditions, for at least 2 h, before starting the experiment. Cells were pre-washed with and kept in HBSS supplemented with 1% (v/v) FCS, 1 mM l-glutamine, 1% (v/v) non-essential amino acids (NEAA), 10 mM *N*-2-hydroxyethylpiperazine-*N*′-2-ethanesulfonic acid (HEPES), 0.55% (w/v) d-glucose while imaging. GUVs were added to the CAR T cell samples and imaged at 37 °C using an incubator stage (Okolab) mounted onto a confocal laser scanning microscope (Nikon Eclipse Ti-E inverted microscope equipped with Nikon A1R confocal laser scanning system, 60 × oil immersion objective, NA = 1.49, with three lasers: 488 nm, 561 nm, and 640 nm). Image acquisition and processing were performed using the NIS-Elements software (version 4.5, Nikon). The assembly of the videos (Online Resource 1–2) was done with Fiji Image J 2.1.0 software.

### Image analysis

The captured time series were analysed for GUVs and CAR T cells contact. First, image segmentation was performed using ilastik [52], and then GUVs, cells and co-localised pixels were quantified. The percent of contact between GUVs and cells was calculated using the following formula: % contact = 100 × (co-localised pixels)/(GUVs pixels + cells pixels). The percentage was estimated for each time point and monitored area.

### Quantification and statistical analysis

Statistical parameters are reported in figures and figure legends, including the exact value of *n*, precision measures (mean ± SEM or median ± SEM) and statistical significance. Data were judged to be statistically significant when *p* < 0.05. In figures, asterisks denote statistical significance as calculated by one-way analysis of variance (ANOVA) or two-way ANOVA as indicated (∗*p* < 0.05; ∗∗*p*< 0.01; ∗∗∗*p* < 0.001; ∗∗∗∗*p* < 0.0001; ns, not significant). Statistical analysis was performed in GraphPad Prism8.

## Results

### Engineered T cells express lectin-based CARs

**Fig. 1:**
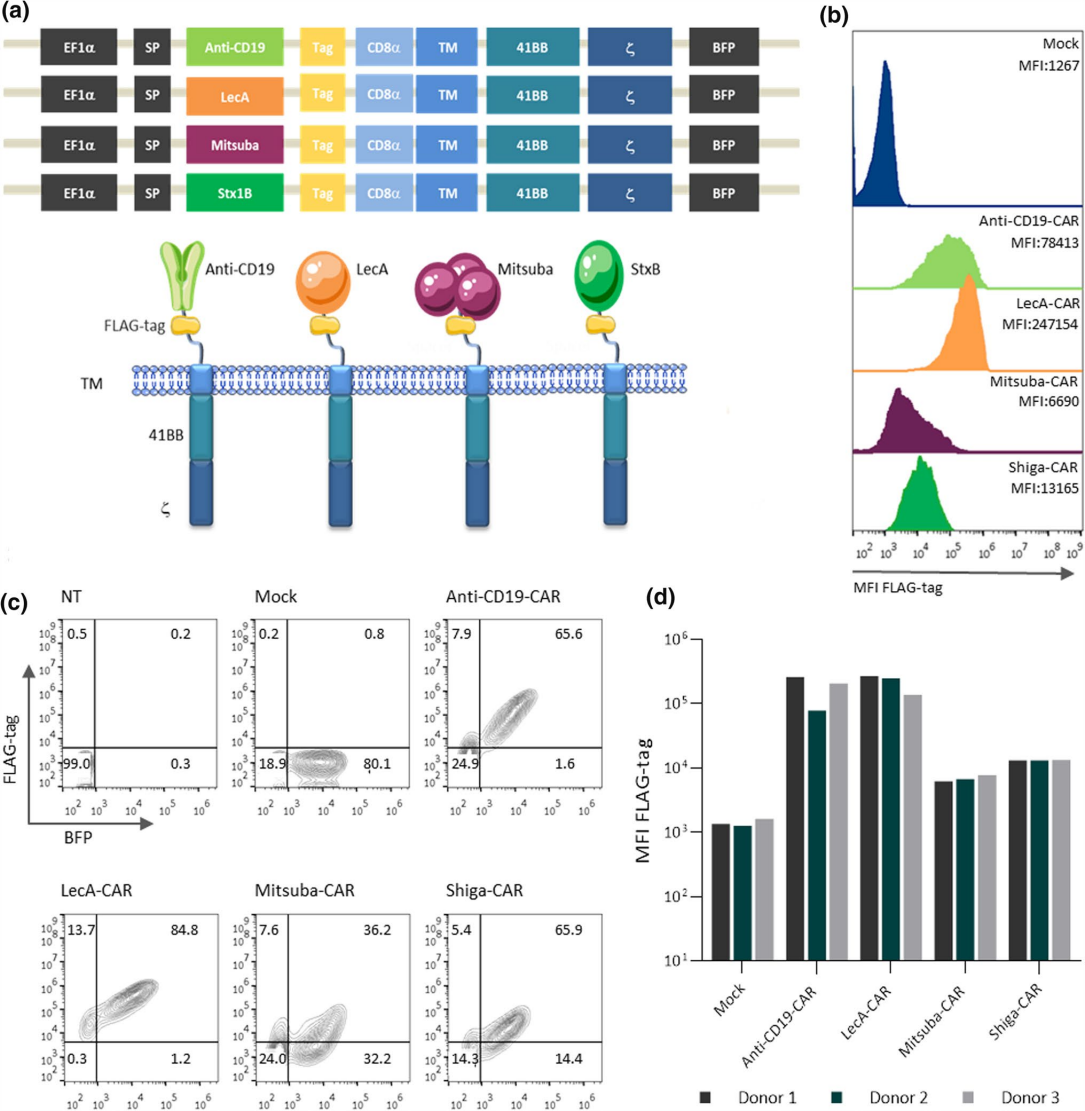
Design of CARs and expression in primary human T cells. **a** Schematic diagram of the lentiviral vector encoding the CARs used in this study. The extracellular domain is tethered to 41BB and the T cell receptor subunit ζ through a transmembrane domain (TM). A T2A peptide sequence separates the CAR from the fluorescent protein BFP. **b–c** Surface expression of CARs and total BFP expression in primary human T cells. PBMCs were transduced with lentiviral vectors encoding CARs and expanded in the presence of IL-2 (100 U mL^−1^). The transduction efficiency was determined by the percentage of BFP^+^ cells and the presence of the FLAG-tag on the cell surface as analysed by flow cytometry. **d** Quantification of the surface expression levels of the five CARs in BFP^+^ cells from three independent healthy donors. Representative data of three independent experiments are shown in b-c

We aimed herein to target Gb3 in tumours with genetically engineered T cells. Previous studies have demonstrated that Gb3 is overexpressed in leukaemia, colorectal, breast, lung, pancreatic, ovarian, and testicular cancer, among other malignancies [53, 54]. Recently, enhanced expression of Gb3 has been described as correlating with the development of metastasis in colon cancer [33, 42] and multi-drug resistance in breast [55], lung, ovarian [56] and myeloid leukaemias [57]. Gb3 was also observed in the neovasculature adjacent to and within the tumor for ovarian carcinomas and their metastases [58], neuroblastomas and astrocytomas [59]. The generation of new vasculature favours tumour development; thus, targeting neovascular cells is an approach to inhibit tumour growth by depriving blood flow. Taken together, these data suggest an exceptionally attractive perspective for the direct targeting of Gb3. However, antibody-mediated targeting of glycosphingolipids appeared difficult [60]. To overcome this limitation, we alternatively designed a novel group of lectin-based CARs. We introduced the sequences of Gb3-binding lectins into a well-characterised second-generation CAR [61]. These novel lectin-CARs have as antigen-binding domain the StxB from *S. dysenteriae*, the LecA from *P. aeruginosa* or Mitsuba, an engineered variant of MytiLec-1 from *M. galloprovincialis* [46]. Sequences encoding these Gb3-binding lectins were cloned in frame into a lentivirus vector to express a second-generation CAR including 41BB and CD3ζ as signalling domains and a fluorescence protein (blue fluorescent protein, BFP) to assay for transduction efficiencies. All CARs in this study share the same transmembrane and signalling domain and include a FLAG-tag for detection (Fig. 1a). As a control, a well-characterised anti-CD19-CAR was used in this study [61].

Activated human PBMCs were lentivirally transduced to express the different lectin-CARs (named Shiga-CAR, LecA-CAR and Mitsuba-CAR), the anti-CD19-CAR or an empty vector, indicated as mock. The transduction efficiency was monitored by BFP expression, and the presence of the CARs on the cell surface was confirmed by FLAG-tag staining and flow cytometry. The BFP expression levels were similar in all cases, indicating similar transduction efficiencies. In contrast, CAR expression levels differed between CARs suggesting differences in folding or transport to the cell surface among the constructs or inaccessibility to the FLAG-tag. The frequency of cells expressing the CARs on the cell surface ranged from 36 to 85% (Fig. 1b, c). The FLAG-tag geometric mean fluorescence intensity (MFI) in CAR T cells revealed that the LecA-CAR had the highest T cell surface expression, followed by anti-CD19-CAR, Shiga-CAR and Mitsuba-CAR, among independent donors (Fig. 1b, d). We next confirmed the CAR expression by immunoblotting (Fig. S1a-b). All CARs exhibited the expected sizes under reducing conditions. In non-reducing gels, higher molecular weight complexes were observed. Despite identical cysteines located in the hinge and transmembrane regions of all CARs, each CAR construct presented a unique oligomerisation pattern (Fig. S1b). The LecA-CAR presented two clear bands that might correspond to a monomeric and tetrameric conformation, which is the native state of LecA. For the Shiga-CAR, two bands were detected, which correspond to that of a dimer or a trimer. The Mistuba-CAR exposed one band corresponding to a monomer. Finally, the anti-CD19-CAR revealed two bands: monomers and higher degree oligomers. Thus, the observed specific pattern of oligomerisation most probably results from the natural tendency of lectins to oligomerise [43]. Each lectin-CAR bears different intrinsic properties, influencing its expression on the cell surface.

### Lectin-based CAR T cells mediate the lysis of tumour cells in vitro

**Fig. 2:**
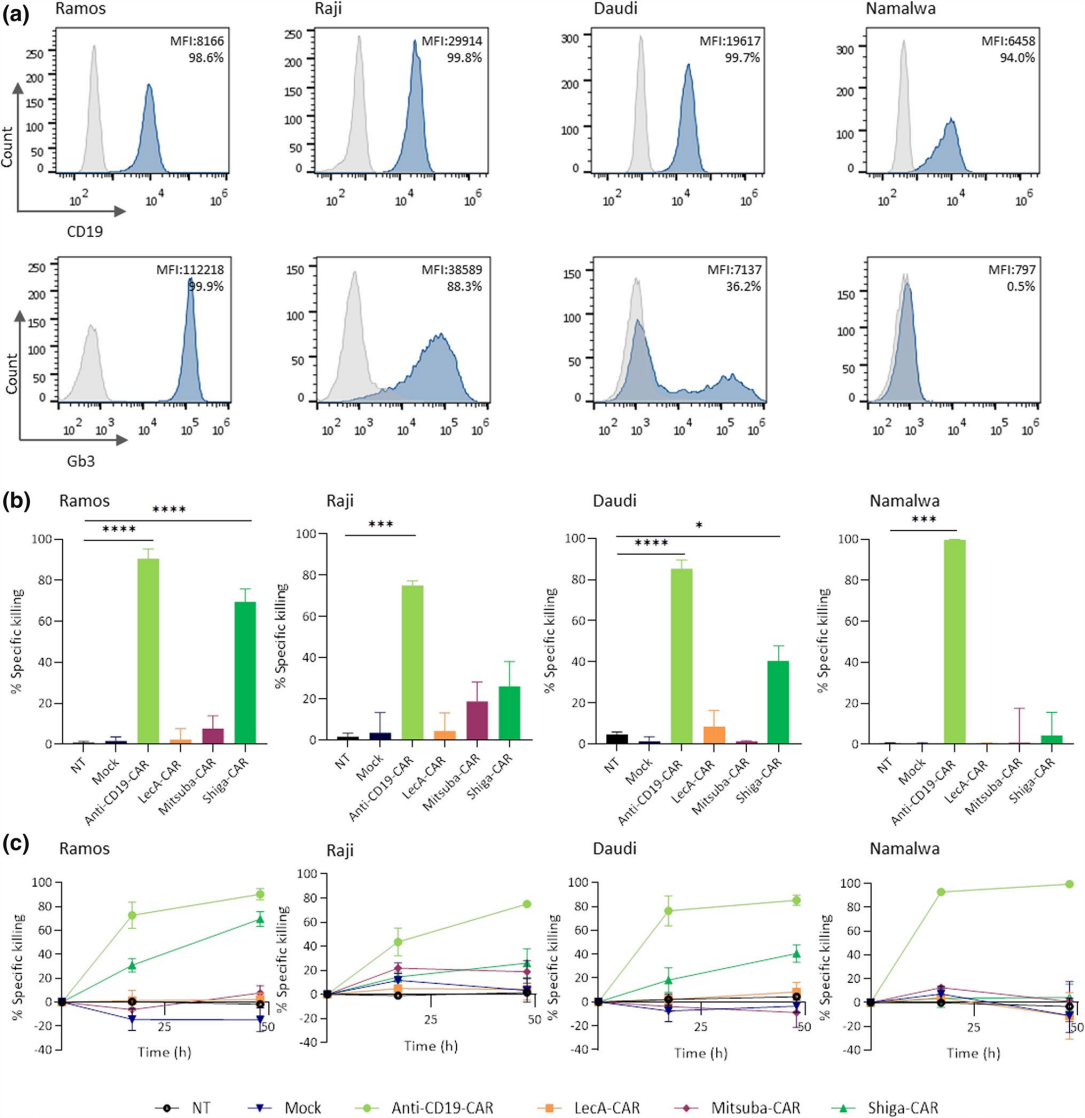
In vitro anti-tumour activity in Burkitt's lymphoma-derived cell lines induced by CAR T cells. **a** Expression of CD19 and Gb3 was assessed by staining with anti-CD19 antibody or labelled StxB and analysed by flow cytometry. Histogram for negative control (grey), CD19 and Gb3 stained samples (dark blue). The values provided in each histogram refer to the geometric mean fluorescence intensity (MFI) and the percentage of positively stained cells. Histograms are representative of at least three independent experiments. **b** Lectin-CARs-specific killing against Burkitt's lymphoma-derived cell lines was checked by luminescence assay. Data show the percent of specific killing after 48 h of co-incubation. **c** Kinetics of the anti-tumour activity of each lectin-CAR against Burkitt's lymphoma cell lines. One-way ANOVA was followed by Dunnett's multiple comparisons test. Mean values ± SEM are shown. *p < 0.05, **p < 0.01 ***p < 0.001, ****p < 0.0001. Data represent at least three independent donors

The anti-tumour functionality of engineered CARs was assayed in a panel of Burkitt's lymphoma-derived cell lines. Cell lines assayed by flow cytometry using fluorescently labelled StxB include three cell lines expressing Gb3 (Ramos, Raji, Daudi) and one cell line that was Gb3^−^ (Namalwa) (Fig. 2a). Importantly, all the cell lines expressed CD19, which is targeted by the anti-CD19-CAR T cells, here used as the positive control. Gb3 expression varied among the cell lines. Ramos cells expressed Gb3 the most (> 99%), followed by Raji (88.3%) and Daudi (36.2%). By contrast, Gb3 was not detected in Namalwa cells. The amount of Gb3 on the cell surface also differed among the studied cell lines (Fig. 2a).

We next co-cultured lectin-CAR T cells with our selection of target cells (Ramos, Raji, Daudi and Namalwa) to assess the efficacy of tumour cell lysis. We implemented a cytotoxicity assay previously described [62] and adjusted the effector to target (E:T) ratio 5:1. We included three controls: activated but untransduced T cells, T cells that were activated and transduced with an empty vector (mock), and T cells that were activated and transduced with an anti-CD19-CAR. Shiga-CAR T cells performed best against all Gb3-expressing tumour cells. In particular, Ramos cells, which presented the highest Gb3 expression, were most efficiently eliminated, followed by Daudi and Raji cells (Figs. 2b, c, S2). After 48 h, the Shiga-CAR T cells achieved 70% specific killing of Ramos cells, and the anti-CD19-CAR T cells 90%, which served as the reference control in this and further experiments. Moreover, Shiga-CAR T cells achieved efficient killing of Gb3^+^ Daudi cells since 36% of Daudi cells were Gb3^+^, and on average, 40% of cells were killed in 48 h (Fig. 2b).

On the contrary, Raji cells seemed to be partially resistant to being targeted with Shiga-CAR T cells regardless expressing high levels of Gb3 (99.8%) among the cells studied. Interestingly, Mitsuba-CAR T cells presented killing against Raji cells (26%, Fig. 2b,c), while the cytotoxicity against Ramos and Daudi was very low (< 7.7%). LecA-CAR T cells displayed poor or no activity against this panel of cell lines. Importantly, none of the lectin-CAR T cells presented activity against the Gb3^−^ Namalwa cells, demonstrating the specificity of the lectin-CARs and the lack of off-target toxicity. Anti-CD19-CAR T cells showed up to 95% cytotoxicity to all the CD19-expressing cell lines after 48 h (Fig. 2b, c).

**Fig. 3:**
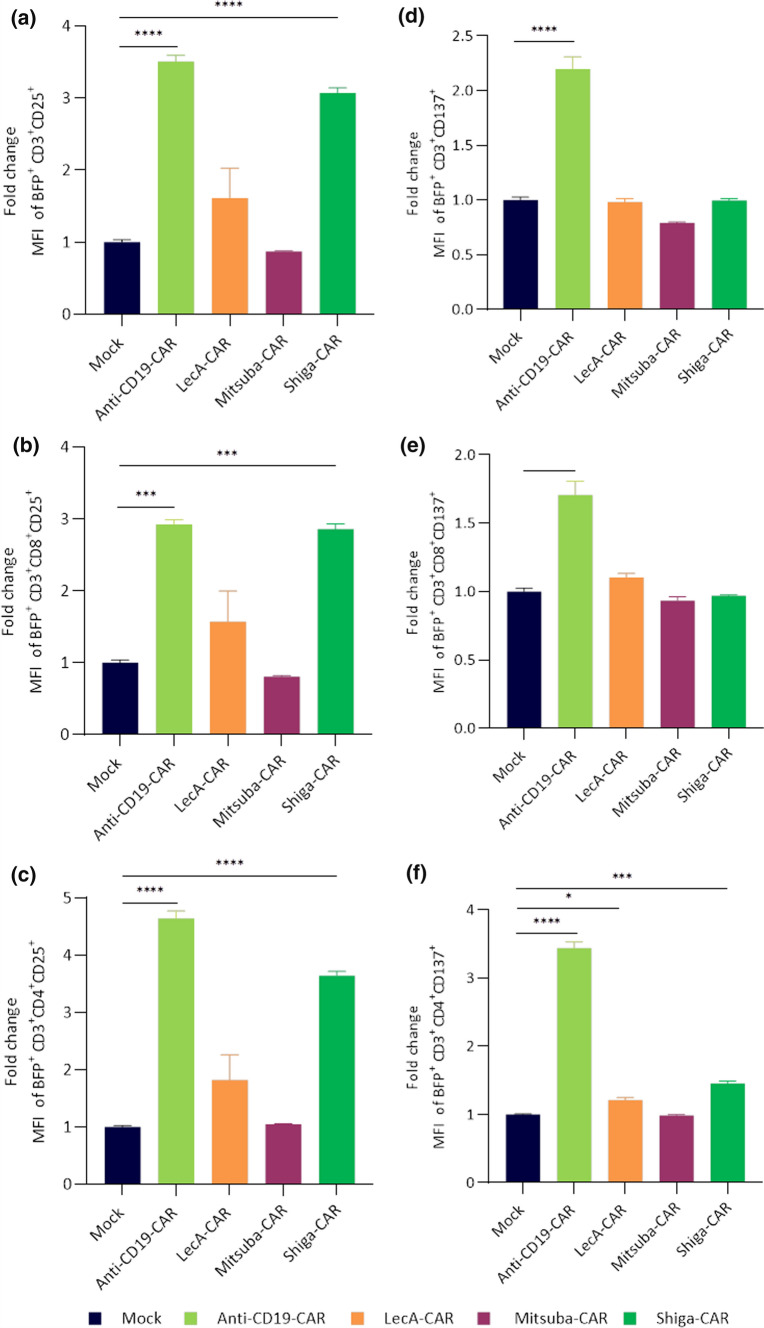
Activation of lectin-CAR T cells. Lectin-CAR T cells were co-incubated with Gb3^+^ Ramos cells for 48 h. Cells were stained with anti-CD3, anti-CD8, anti-CD25 and anti-CD137 antibodies and analysed by flow cytometry. The MFI value of each subpopulation was normalised to the corresponding mock to determine the fold change. Fold change in CD25 expression of lectin-CAR T cell subpopulations. **a** Total T cells, BFP^+^CD3^+^CD25^+^, **b** cytotoxic CD8 T cells, BFP^+^CD3^+^CD8^+^CD25^+^, **c** helper CD4 T cells, BFP^+^CD3^+^CD4^+^CD25^+^. Fold change in CD137 expression of lectin-CAR T cell subpopulations gated as before **d** total T cells, **e** cytotoxic CD8 T cells and **f** helper CD4 T cells. One-way and two-way ANOVA followed by Dunnett's multiple comparisons test. Mean values ± SEM are shown. *n* = 3, simultaneously performed stimulations. *p < 0.05, **p < 0.01 ***p < 0.001, ****p < 0.0001. Representative data of one out of two independent donors

T cells must actively and specifically recognise foreign antigens to mount an effective immune response. The antigen-induced signal over tonic signal can be used to determine effective T cell stimulation. Thus, we next analysed lectin-CAR T cell activation upon co-culturing with Gb3^+^ and CD19^+^ Ramos cells in a 5:1 effector to target cells ratio for 48 h. We then determined the expression of the T cell activation markers CD137 and CD25 in BFP^+^CD3^+^ lectin-CAR T cells by flow cytometric analysis. Both CD8^+^ and CD4^+^ Shiga-CAR T cells displayed a substantial and significant increase in CD25 expression after exposure to Gb3^+^ Ramos cells (Fig. 3a–c). CD8^+^ and CD4^+^ LecA-CAR T cells slightly increased CD25 expression upon contact with Gb3^+^ Ramos cells, while Mitsuba-CAR T cells failed to recognise or establish a sustained binding to target cells and maintained similar CD25 expression compared to mock cells. Anti-CD19-CAR T cells exhibited strong CD25 expression after CD19 stimulation (Fig. 3a–c).

Conversely to CD25, none of the lectin-CAR T cells upregulated CD137 upon contact with Gb3^+^ Ramos cells (Fig. 3d–f). These results are in sharp contrast with the behaviour of the anti-CD19-CAR T cells, which strongly increased the expression of CD137 upon incubation with CD19^+^ Ramos cells (Fig. 3d–f). Despite having identical signalling domains, these data suggest differences in signal strength and quality between the lectin-CARs and the anti-CD19 CAR. Differential behaviour could be explained mechanistically by differences in clustering geometry, ligand density, affinity or all of them.

### Improving activation and cytotoxicity of lectin-CAR T cells

**Fig. 4:**
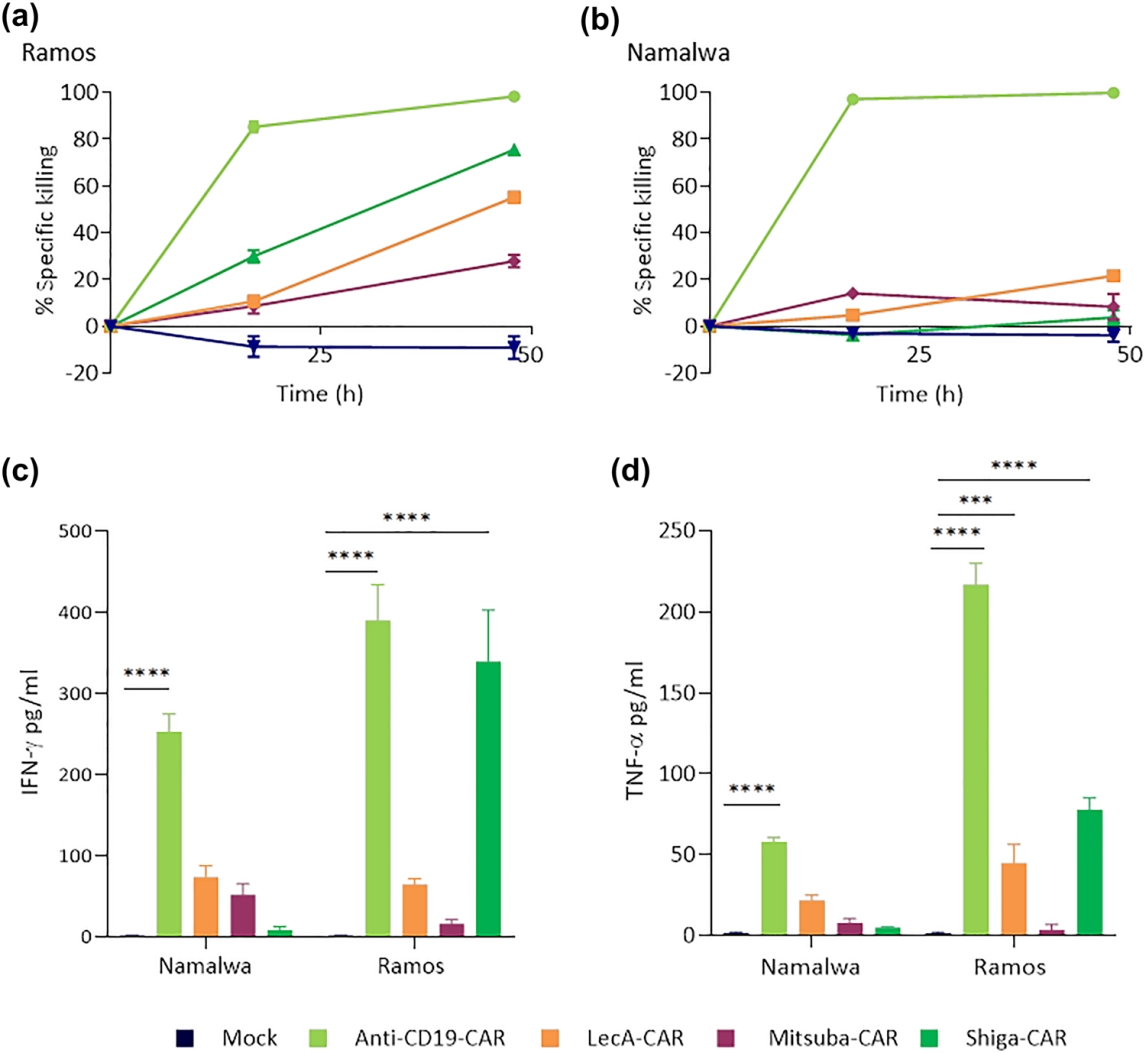
In vitro cell cytotoxicity and cytokine secretion by lectin-CAR T cells. **a** Kinetics of killing by BFP-sorted lectin-CAR T cells towards Gb3^+^ CD19^+^ Ramos and Gb3^−^ CD19^+^ Namalwa cell lines. Results were normalised to mock. Amount of IFN-γ (**b**) and TNF-α (**c**) secreted by CAR T cells after 48 h of co-incubation with the indicated target cells. Effector to target (E:T) cells ratio was set to 5:1. Effector CAR T cells were BFP-sorted. Two-way ANOVA was followed by Dunnett's multiple comparisons test. Mean values ± SEM are shown. *p < 0.05, **p < 0.01, ***p < 0.001, ****p < 0.0001. *n* = 3, simultaneously performed co-incubations. The data shown here were normalised to mock, as the cells were subjected to the same conditions during and after sorting. Representative data of one experiment out of two independent donors

To assess potential confounding effects imparted by the different proportions of primary T cells expressing each lectin-CAR, we sorted BFP^+^ cells to obtain homogeneous transduced populations. Upon sorting, cells were maintained in culture for 48 h to recover. The sorted CAR T cells were then co-incubated with Gb3^+^ Ramos and Gb3^−^ Namalwa cells, as described earlier. After 48 h, Shiga-CAR T cells achieved 75% of specific killing against Ramos cells, similar to unsorted cells, 69.6% (Figs. 2b, 4a). Surprisingly, enrichment of Mitsuba-CAR T cells and LecA-CAR T cells allowed a remarkable increase in anti-tumour activity compared to unsorted cells (7.7% vs 27.8% and 2.2% vs 55.%, respectively; Figs. 2b, 4a), demonstrating that these CAR constructs were indeed functional. Moreover, sorting of the lectin-CAR T cells did not alter their specificity since their cytotoxic activity towards Namalwa cells was very poor or absent. The cytotoxicity of sorted anti-CD19-CAR T cells against Ramos and Namalwa was slightly higher compared to unsorted cells (90.3% (unsorted) vs 98.3% (sorted); Figs. 2b, 4a). Taken together, sorted lectin-CAR T cells were accurately recognised and killed Gb3^+^ cells lacking undesired off-target effects. The Shiga-CAR T cells were the best in targeting Gb3^+^ cells among the lectin-CAR T cells.

One of the hallmarks of activated T lymphocytes is the production of cytokines, which in turn activate efficient immune responses. In contrast to anti-CD19-CAR, lectin-CARs efficiently drove upregulation of CD25 but not of CD137 (Fig. 3). Therefore, it was essential to evaluate whether lectin-CARs induced the secretion of cytokines. We co-cultured BFP^+^-sorted cells with Ramos or Namalwa cells for 48 h and collected the supernatants to analyse cytokine release by ELISA. We tested the secretion of IFN-γ and TNF-α in response to co-cultures with Gb3^+^ Ramos cells and Gb3^−^ Namalwa cells. Shiga-CAR T cells efficiently and specifically secreted IFN-γ to the supernatant upon incubation with Ramos leukaemia cells (340 pg mL^−1^, Fig. 4b) compared to incubation with Gb3^−^ Namalwa cells (Fig. 4b). However, LecA-CAR and Mitsuba-CAR T cells did not secrete IFN-γ above the background levels when stimulated with Gb3^+^ Ramos or Gb3^−^ Namalwa cells (Fig. 4b). The anti-CD19-CAR T cells released IFN-γ to the same extent as Shiga-CAR T cells when incubated with Ramos cells (390 pg mL^−1^, Fig. 4b). TNF-α secretion by lectin-CAR T cells that were co-incubated with Gb3^+^ Ramos cells was lower, 78 pg mL^−1^ for the Shiga-CAR, 45 pg mL^−1^ for the LecA-CAR T cells, and Mitsuba-CAR T cells failed to produce TNF-α (Fig. 4c). TNF-α secretion by Shiga-CAR T cells was specific to recognising Gb3 since it was absent upon incubation with Gb3^−^ Namalwa leukaemia cells. Anti-CD19-CAR T cells released considerably higher amounts of TNF-α (217 pg mL^−1^) upon incubation with Ramos cells. The affinity of the antigen binding domain can influence TNF-α release. Indeed, lowering the affinity of an anti-CD19 scFv led to lower TNF-α release in a recent study. The anti-CD19 scFv FMC63 used in this study has a binding affinity in the nanomolar order [63], while the affinity/avidity of the lectins for Gb3 ranges from the nanomolar to milimolar order [64]; these differences may help to understand disparities in TNF-α release.

In summary, these data indicate that Shiga-CAR T cells specifically produced cytokines upon recognition of Gb3 on the surface of target leukaemic cells. While Shiga-CAR T cells secreted as much IFN-γ as anti-CD19-CAR T cells, they secreted less TNF-α, which could be indeed beneficial to reduce the risk of cytokine release syndrome, a well-described life-threatening secondary effect in patients treated with anti-CD19-CAR T cells [65, 66].

### Specificity of lectin-CAR T cells

**Fig. 5:**
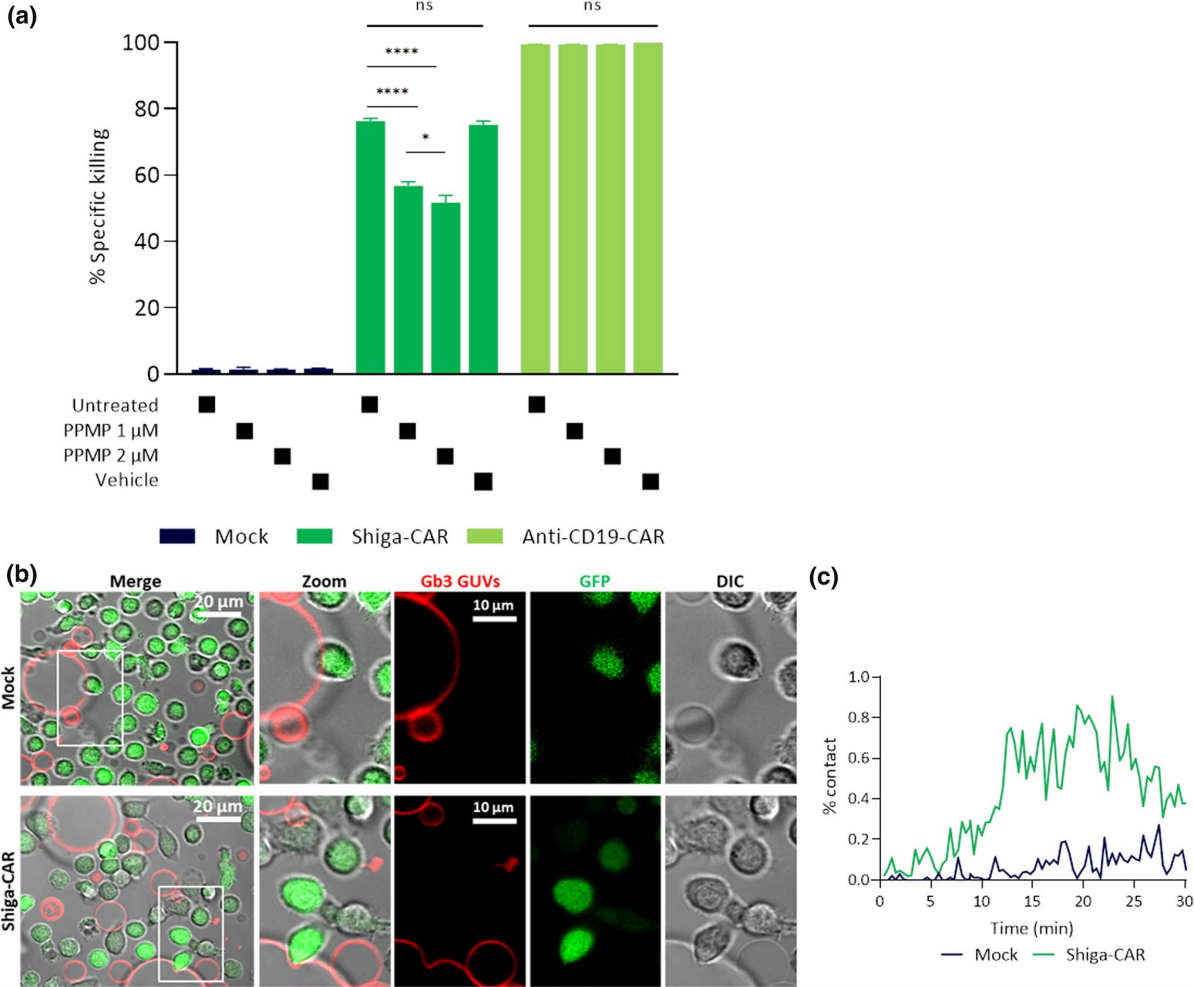
Gb3 is specifically recognised by Shiga-CAR T cells. **a** Ramos leukaemic cells were treated as indicated and co-incubated for 24 h with effector T cells. Two-way ANOVA was followed by Dunnett's multiple comparisons test. Mean values ± SEM are shown, n = 3, simultaneously performed co-incubation. *p < 0.05, **p < 0.01 ***p < 0.001, ****p < 0.0001, ns, not significant. Representative data of one out of two independent donors. **b** Live cell imaging of mock and Shiga-CAR T cells, which were co-incubated with giant unilamellar vesicles (GUVs) decorated with Gb3. Shiga-CAR T cells in green and GUVs in far-red. GUVs were composed of DOPC, cholesterol, Atto 647N DOPE and Gb3 (59.7:30:0.3:10 mol%, respectively). Images from selected areas were monitored. Zoom areas are marked with a white frame, and the arrow indicates membrane bending. Scale bars  10 and 20 µm **c** The contact of Shiga-CAR T cells or mock t cells and GUVs is displayed as a percent. Quantified green, red and co-localised pixels were used to calculate the percent of contact. Lines represent median values of the percent of contact in the monitored areas over time

Shiga-CAR T cells displayed the best anti-tumour activity against high Gb3^+^ Ramos leukaemic cells. To formally demonstrate that the Shiga-CAR T cells specifically target Gb3, Ramos cells were treated with PPMP to inhibit the synthesis of glucosyl-ceramide-based glycosphingolipids. PPMP is a ceramide analogue designed to inhibit the glycosyltransferase involved in the biosynthesis of glucosylceramides [67], primary reducing the expression of Gb3 [103]. Ramos cells were exposed to 1 µM and 2 µM of PPMP for 3 days. On the third day, the levels of Gb3 on the cell surface were assessed by staining the cells with fluorescently labelled StxB. Flow cytometric analysis showed that PPMP treatment efficiently reduced the levels of Gb3 on the cell surface of Ramos cells, similar to unstained cells (Fig. S3a). Cells treated with PPMP or vehicle (dimethyl sulfoxide, DMSO) were co-cultured with Shiga-CAR T cells and controls, mock and anti-CD19-CAR T cells. PPMP-treated Ramos cells were significantly less killed by Shiga-CAR T cells after 12 h (36%, 1 µM, and 26%, 2 µM) and 24 h (54% and 46%, respectively) compared to untreated or vehicle-treated Ramos cells (both > 56% at 12 h and both > 75% at 24 h, Fig. 5a, and Fig. S3b). The reduction was dose-dependent with decreased cytotoxicity at higher concentrations of PPMP. Cytotoxicity was detected to some extent, as it cannot be excluded that Gb3 is present on cell surface at levels that are below the detection threshold in flow cytometry, but still being recognised by Shiga-CAR T cells. Furthermore, the treatment with PPMP did not affect the viability of Ramos cells or the presence of CARs on the T cell surface (Fig. S3c-d), and the cytotoxic activity of controls, mock and anti-CD19-CAR T cells, remained unaffected.

To further support the specificity of the lectin-CAR T cells, we transfected the non-tumorigenic Madin-Darby canine kidney (MDCK) cell line with Gb3 synthase (α1,4-galactosyltransferase), creating MDCK Gb3^+^ cells and comparing them to their wild type counterparts (MDCK WT). Flow cytometry analysis revealed that the MDCK Gb3^+^ cells heterogeneously expressed Gb3, with 62% positive cells (Fig. S4a), while Gb3 was not detected in the parental MDCK WT cells (Fig. S4b). The Shiga-CAR T cells efficiently killed MDCK Gb3^+^ cells (Fig. S4c); at 17 h, the Shiga-CAR T cells reached 54% killing. In contrast, the MDCK WT cells were not targeted (Fig. S4d). Taken together, these data strongly argue for a specific cytotoxic effect following Gb3-dependent activation of lectin-CARs.

### Gb3 recognition in cell-sized giant unilamellar vesicles by Shiga-CAR T cells

Next, we applied a simplified and controlled membrane system to demonstrate that the presence of Gb3 is sufficient for recognition by Shiga-CAR T cells. Giant unilamellar vesicles (GUVs) are synthetic cell-sized spherical lipid bilayers resembling the size of human cells that can be glyco-decorated with diverse glycans [68, 69]. They have been explored to rebuild and, therefore, to better understand cellular processes, e.g., adhesion [70] and endocytosis [68, 71]. We produced GUVs containing 10 mol% Gb3 within the chosen lipid mixture (DOPC, cholesterol, Atto 647N-DOPE, and Gb3; 59.7:30:0.3:10 mol%, respectively) by electroformation [51]. On the day of the experiment, sorted Shiga-CAR T cells or mock T cells were stained with the CellTrace™ CFSE reagent for visualisation by confocal microscopy. The GUVs were visible in far-red and CAR T cells in green. GUVs and T cells were co-incubated and monitored for 30 min at 37 °C. We acquired distinct areas using a live cell imaging setting by confocal microscopy. The Shiga-CAR T cells and mock T cells continuously approached GUVs and surrounded them. The interactions between Shiga-CAR T cells and GUVs were more likely than with mock T cells (Online Resource-1–2). Over time, more and more cells enriched the areas around the GUVs as trying to build contacts. In some cases, the Gb3-containing GUVs were deformed exclusively during the interactions with Shiga-CAR T cells. Such events were not observed upon incubation with mock cells (Fig. 5b). Inward bending of Gb3^+^ GUVs probably indicates tight interactions of Shiga-CAR T cells with Gb3-functionalised vesicles, related to membrane invaginations induced by StxB [72, 73] or LecA in Gb3^+^ GUVs and HEK cells [44, 74]. More recently, a molecular dynamics simulation also proposed the induction of negative membrane curvature triggered by StxB and LecA after binding to and clustering Gb3 [75].

We analysed the contact between GUVs and T cells to quantify these interactions using the Ilastik software [52]. For this purpose, a protocol for machine learning was developed, and time series were analysed after image segmentation. The contact between GUVs and cells was estimated using the percent of contact. The quantification of pixels in green (cells), red (GUVs), and co-localised were considered to calculate the percent of contact, which was determined for each monitored area and time point. The analysis revealed cell interactions in both conditions, but the percent of contact was higher for the Shiga-CAR T cells than for the mock T cells (Fig. 5c). These results suggest that Gb3 drives the interactions with the Shiga-CAR T cells. This interaction might occur independently of any additional protein on the surface of target cells, as we used a synthetic system lacking additional proteins or active cellular machinery.

As briefly mentioned above, the interactions between the homopentameric StxB and Gb3 in synthetic membrane systems and living cells are well documented in the literature [72, 76–78]. Considering that only a single StxB monomer was part of the CAR construct, it was somewhat surprising to observe that StxB monomers in the framework of CARs might facilitate these interactions. Additionally, Shiga-CARs showed dimers and trimers in primary T cells (Fig. S1b), which might increase the avidity for Gb3 on the GUV and host cell membrane. Taken together, we successfully demonstrated that Gb3 and the GUV environment were sufficient to drive the interactions with the carbohydrate-binding domain of the Shiga-CAR, strongly supporting the selectivity of our approach.

### Lectin-CAR T cells efficiently target solid tumour cell lines

**Fig. 6:**
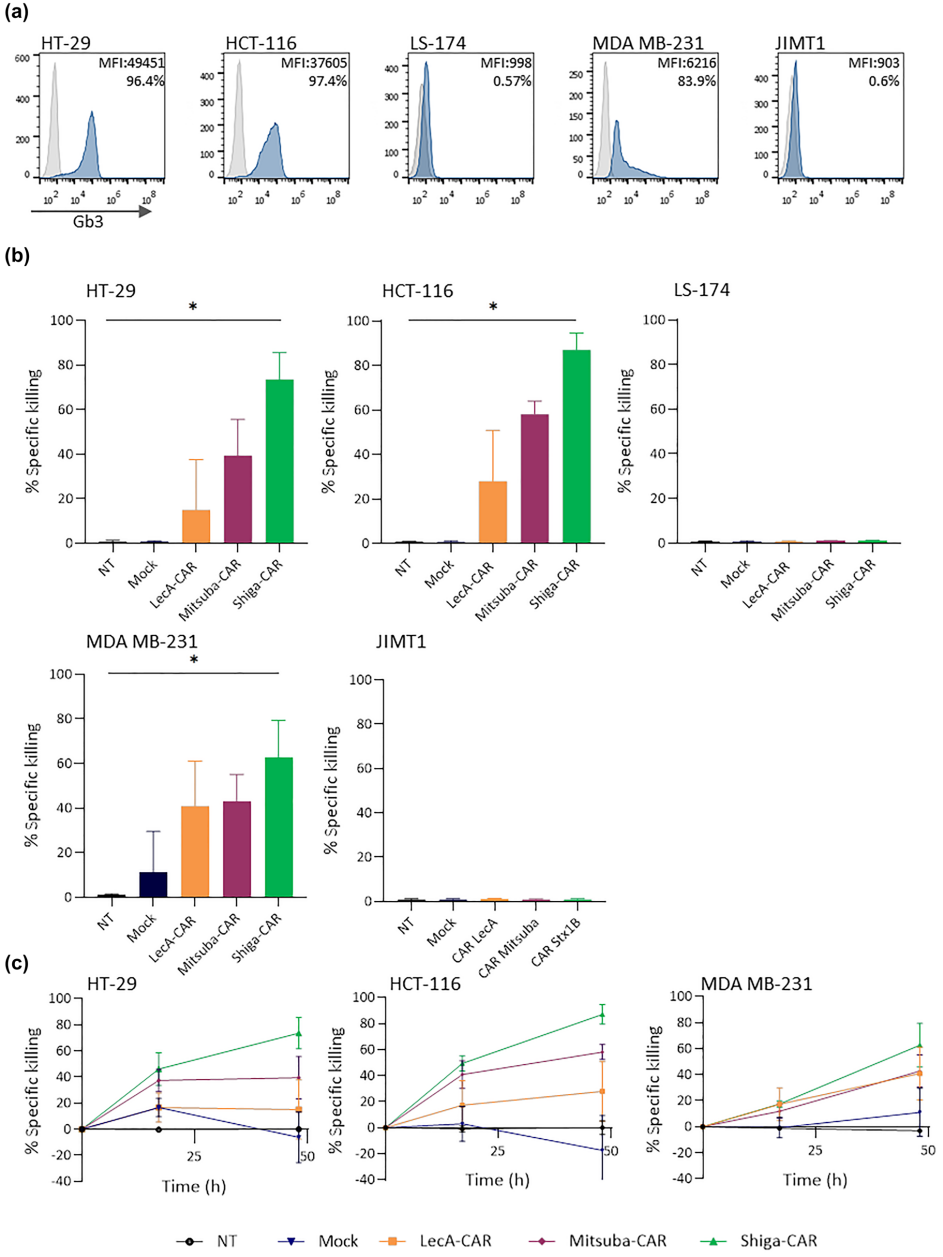
In vitro solid tumour cell killing by CAR T cells. **a** Expression of Gb3 was assessed by staining with labelled StxB and flow cytometry. **b** Lectin-CAR specific killing of colorectal cancer cell lines (HT-29, HTC-116, LS-174) and breast cancer cell lines (MDA-MB-231 and JIMT1) was checked by a bioluminescence assay at 48 h. One-way ANOVA was followed by Dunnett's multiple comparisons test. Mean values ± SEM are shown. Data represent at least three independent experiments. *p < 0.05, **p < 0.01, ***p < 0.001, ****p < 0.0001. **c** Kinetics of the killing of each lectin-CAR against solid tumour cell lines. Mean values ± SEM are shown. Data represent at least three independent experiments

One major obstacle for using CAR T cell technology against solid tumours is the lack of tumour-specific antigens [79–81]. Since Gb3 is overexpressed in colorectal carcinoma [33], breast [34], ovary and pancreatic cancer [35], as well as gliomas [36], we decided to test the anti-tumour activity of the lectin-CARs against cell lines derived from colon and breast cancer. First, we checked the expression of Gb3 in a panel of cells by staining them with fluorescently labelled StxB (Fig. 6a). Flow cytometry analysis revealed that colorectal cancer cell lines HT-29 and HCT-166 expressed Gb3 (> 95% positive cells). MDA-MB-231 cells heterogeneously expressed Gb3, with 83% positive cells overall. In contrast, Gb3 was not detected in LS-174 colorectal cancer cell and JIMT1 breast cancer cell lines.

Next, we performed cytotoxic assays using our complete panel of primary lectin-CAR T cells. Similar to the results obtained for leukaemia cells, Shiga-CAR was the best at targeting Gb3 (Fig. 6b, c and Fig. S7). Shiga-CAR T cells showed the highest anti-tumour activity against HT-29 (74%) and HCT-166 (90%) colorectal cancer cells and MDA-MB-231 triple-negative breast tumour cells (60%). The possibility of targeting triple-negative breast cancer cells with lectin-CAR T cells is remarkable. Triple-negative breast cancer is the most aggressive and lethal breast cancer subtype, for which no targeted treatment options are currently available [82]. Mitsuba-CAR T cells displayed cytotoxic activities varying between 40 and 60% among the cell lines expressing Gb3, namely HT-29, HCT-166 and MDA-MB-231. Meanwhile, the cytotoxicity exhibited by LecA-CAR T cells was the lowest (10–24%) compared to other lectin-CARs and showed a high intrinsic variation among individual donors. Taken together, lectin-CARs efficiently targeted Gb3^+^ colorectal and breast cancer cells while lacking cytotoxicity against Gb3^−^ tumour cells, further supporting the specificity of Gb3 recognition. The recognition of cell lines derived from solid tumours by lectin-CAR T cells appears to be even more efficient than the recognition of leukaemic cells.

**Fig. 7:**
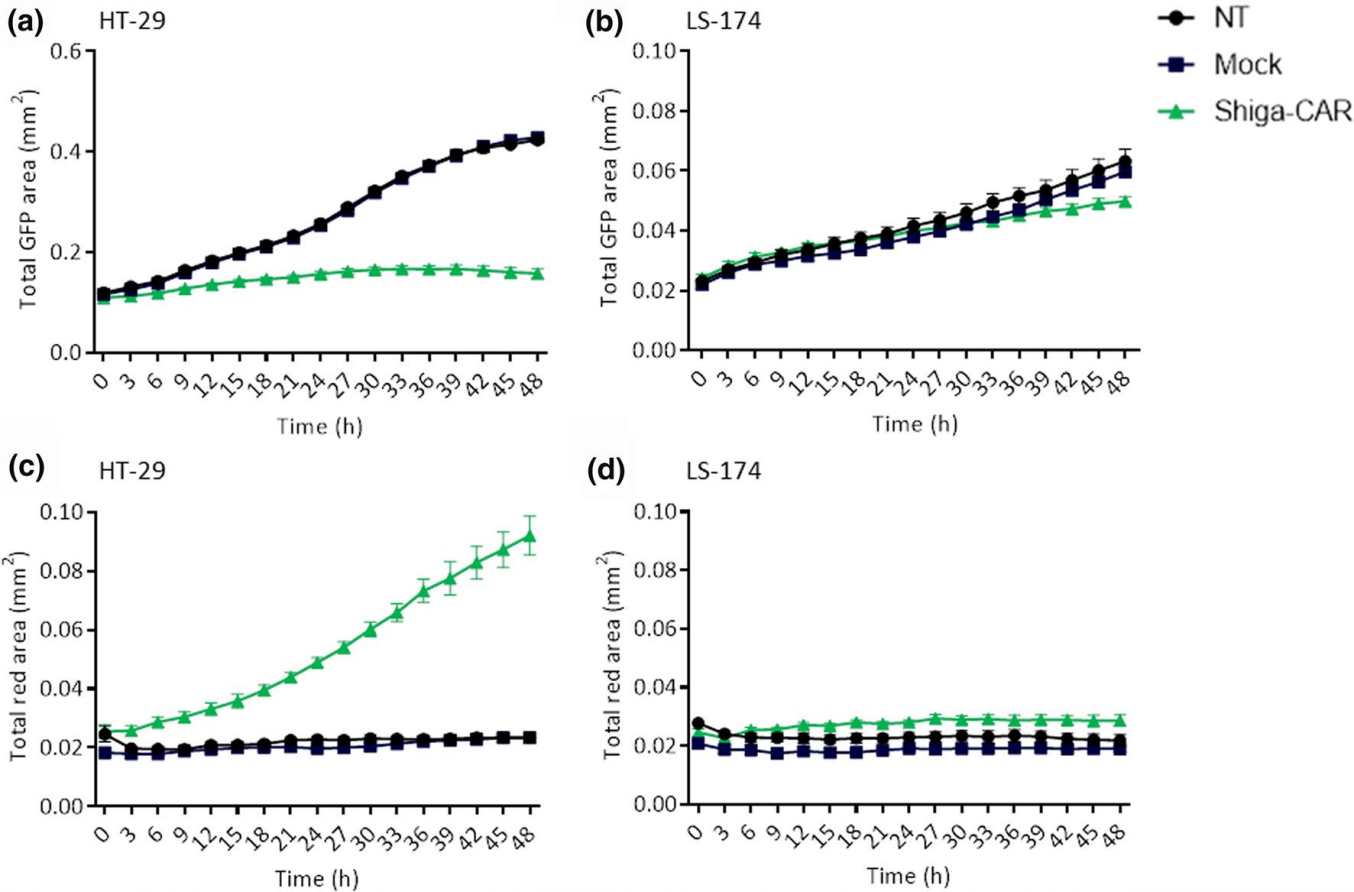
Live imaging of lectin-CAR T cells cytotoxicity. A cytotoxicity assay was performed using GFP^+^ Gb3^+^ HT-29 and the GFP^+^ Gb3^−^ LS-174 colon cancer cells and monitored by live cell imaging. **a, b** Data show cellular proliferation of GFP^+^ cells up to 48 h, images were taken every 3 h. **c, d** HT-29 and LS-174 cells were stained with C.LIVE Tox Red, a sensitive dye that binds to DNA. The dye enters dead cells through damaged plasma membranes and emits fluorescence after binding. We monitored cell fluorescence for 48 h, taking images of all wells every 3 h. Total area in green or red was quantified. Mean values ± SEM are shown. *n* = 4, simultaneously performed co-incubations, representative data of one experiment out of two independent donors

Furthermore, we have checked the killing activity of the Shiga-CAR T cells in Gb3^+^ GFP^+^ HT-29 and Gb3^−^ GFP^+^ LS-174 colon cancer cell lines using live cell imaging. On the one hand, we monitored the total amount of cells by quantifying the GFP signal and determining the total GFP area. On the other hand, we stained the cells with C.LIVE Tox Red, a sensitive dye that binds to DNA, which is non-permeable to viable cells and non-fluorescent outside of the cells. After entering dead cells through damaged plasma membranes and binding to DNA, the reagent emits a bright red fluorescence. We monitored the GFP and C.LIVE Tox Red signals of the cells with live cell imaging for 48 h, taking images every 3 h. Shiga-CAR T cells drastically reduced the numbers of HT-29 cells (Figs. 7a, S5a), whereas the LS-174 cells, which are Gb3^−^, continued growing (Figs. 7b, S6a). The NT and mock T cells did not affect cell proliferation in either cell line. The red fluorescence of the HT-29 cells, indicative of cell death, increased when co-incubated with Shiga-CAR T cells (Figs. 7c, S5b). In contrast, the red signal from the LS-174 Gb3^−^ cells remained unaffected (Figs. 7d, S6b). NT and mock T cells did not trigger fluorescence changes in both cell lines. This live image analysis allowed us to observe that the proliferation of HT-29 cells slowed down between 18 and 21 h (Fig. 7a), while the Tox Red signal notably increased whitin this time window (Fig. 7c). HT-29 cells continued proliferating when co-incubated with NT and Mock cells (Fig. S5a), instead they were almost completely eliminated after 48 h when co-incubated with Shiga-CAR T cells (Fig. S5b). Taken all together, the results strongly uphold the killing ability and specificity of the Shiga-CAR T cells.

**Fig. 8:**
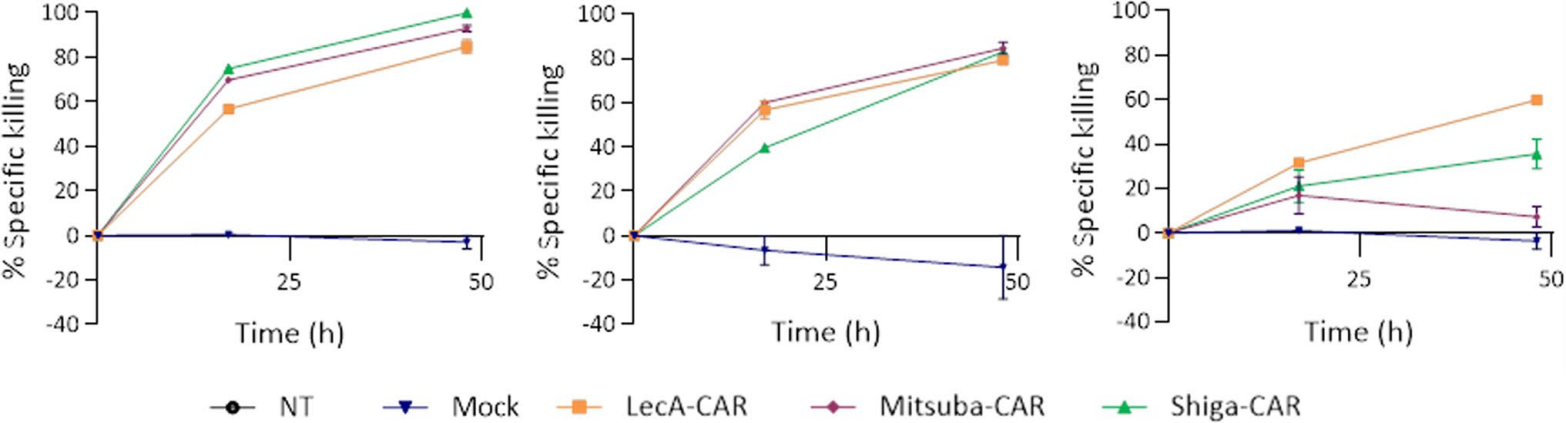
Killing kinetics of BFP-sorted lectin-CAR T cells. Mean values ± SEM are shown. *n* = 3, simultaneously performed co-incubations. Mock was considered for normalisation as the cells were subjected to the same conditions during and after sorting. Representative experiment out of two independent donors

Next, we sorted BFP^+^ CAR T cells and investigated whether the killing activity could be enhanced when working with pure CAR T cell populations as previously observed (Fig. 4). Using pure populations, Mitsuba-CAR (92% and 84%) and LecA-CAR T cells (85% and 79%) achieved similar killing activity as Shiga-CAR T cells (99.8% and 83%) when co-incubated with HT-29 and HCT-116 tumour cells (Fig. 8). In this way, anti-tumour activity was also enhanced, and the percentage of killed cells increased at the endpoint of the experiment. The anti-tumour activity of the LecA-CAR and Mitsuba-CAR in solid tumours appears to be vastly improved. The performance of lectin-CARs against cell lines derived from colorectal and breast cancer is promising, considering the lack of cancer-specific targets.

### Lectin-CARs recognise a wide variety of Gb3 isoforms

**Fig. 9:**
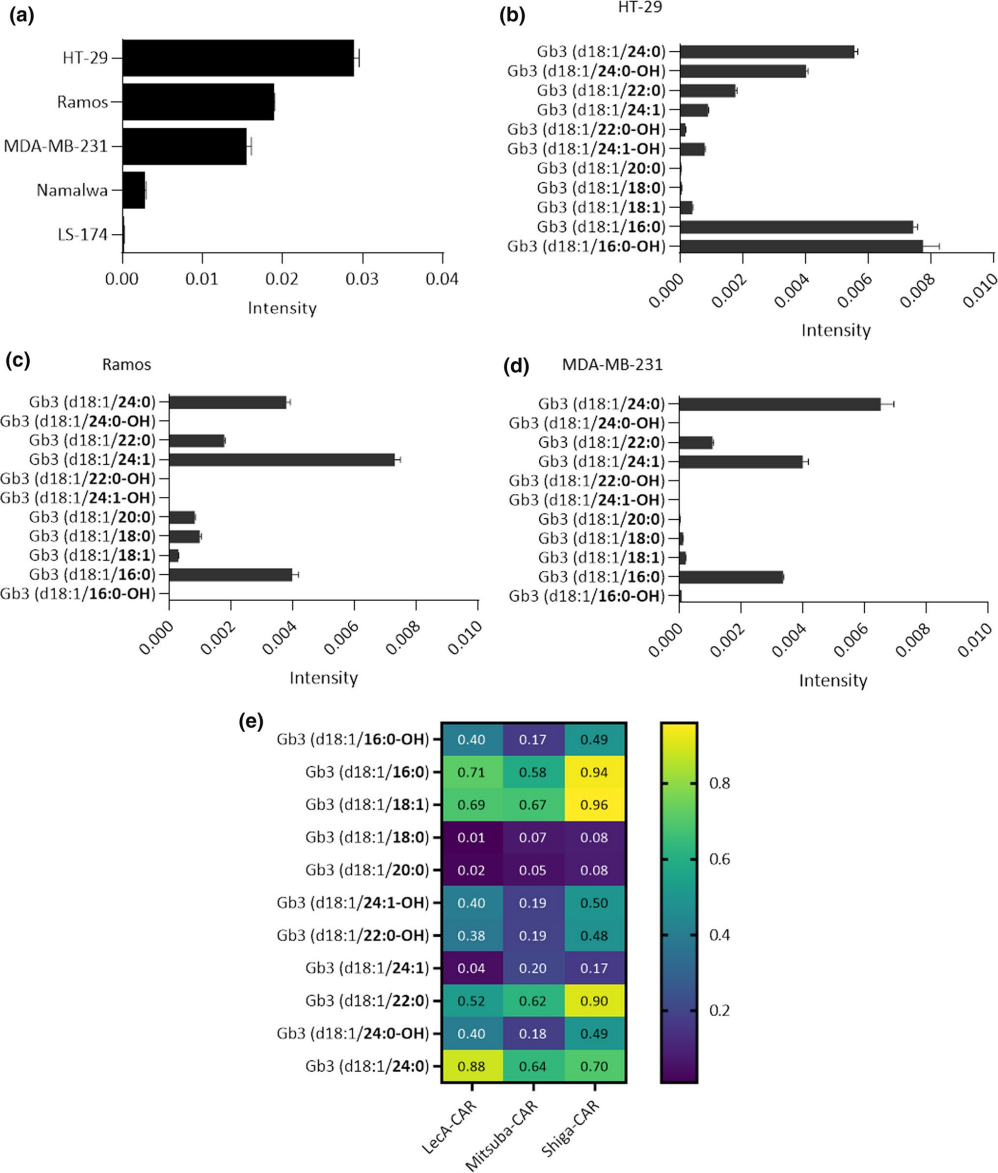
Lipid analysis by MS reveals Gb3 abundance and dominant isoforms present in the studied cell lines. **a** Total Gb3 content per cell line. **b** Distribution of Gb3 isoforms in HT-29 cells. Most common present Gb3 isoforms were (d18:1/C16:0-OH), (d18:1/C16:0) and (d18:1/C24:0). **c** Distribution of Gb3 isoforms in Ramos cells. Most abundant Gb3 isoforms were (d18:1/C24:1), (d18:1/C16:0) and (d18:1/C24:0). **d** Distribution of Gb3 isoforms in MDA-MB-231 cells. Dominantly present species were (d18:1/C24:0), (d18:1/C24:1) and (d18:1/C16:0). Displayed Gb3 species were normalised to an internal standard and the sum of all detected sphingolipids. Mean values ± SEM are shown. *n* = 3, analysed samples in one LC–MS sequence. **e** Heat map of Pearson's correlation coefficient of the Gb3 isoform abundance and the specific killing by lectin-CAR T cells

Membrane lipids are not homogenously distributed, leading to membrane domains of specific composition and order [83, 84]. Glycosphingolipids so do Gb3 segregates into different domains. The membrane microenvironment and the Gb3 fatty acyl chains determine how Gb3 molecules are embedded in the lipid bilayer and, consequently, how the carbohydrate head of Gb3 will be exposed [30, 85, 86]. StxB and LecA have shown preference to recognise Gb3 isoforms distributed in different membrane domains in which the carbohydrate head group of Gb3 is differently exposed. [76]. Therefore, to better understand the influence of Gb3 abundance and diversity on the recognition by lectin-CAR T cells, we characterised the amount of Gb3 isoforms, which can vary in fatty acyl chain length, degree of saturation and hydroxylation, in several of the target cells used in this study (HT-29, Ramos, MDA-MB-231, Namalwa and LS-174). Cells were lysed in a mixture of methanol and water 1:1, followed by chloroform purification, allowing lipid isolation for later targeted liquid chromatography and mass spectrometry. The normalised intensities indicate the total abundance of Gb3 (Fig. 9a). HT-29 cells (0.028) exhibited the highest total amount of Gb3, followed by Ramos (0.018) and MDA-MB-231 (0.015) cells. Namalwa (0.002) had little Gb3 content, and Gb3 was not detected in LS-174 cells (Fig. 9a), confirming our flow cytometric data (Fig. 2a and Fig. 6a). We next performed a Pearson correlation between the total Gb3 abundance detected by MS analysis in each cell line and the cytotoxicity of the lectin-CAR T cells. We aimed to understand whether the abundance of Gb3 influences the efficacy of the lectin-CAR T cells in lysing cells. The analysis revealed a solid Gb3 dependency for the Shiga-CAR T cell-induced cytotoxicity (*r*_Shiga-CAR_ = 0.9742) and a moderate dependency for LecA-CAR T cells (*r*_LecA-CAR_ = 0.7274) and Mitsuba-CAR T cells (*r*_Mitsuba-CAR_ = 0.6197). In general, the higher is the abundance of Gb3 on the target cells, the higher is the cytotoxicity (Fig. S7). However, a deeper analysis of the dominantly present Gb3 species revealed that the studied model cells lines contain many Gb3 isoforms. In HT-29 colorectal cancer cells, the isoform C16:0-OH and C16:0 each made up 25% of the Gb3 species present. The saturated species C24:0 represented 19%, and its hydroxylated isoform C24:0-OH 13%. Additionally, 6% of C22:0 and small traces of C18:1; C18:0, C20:0, C24:1-OH, C22:0-OH and C24:1 were detected (Fig. 9b). Interestingly, hydroxylated Gb3 isoforms, which have been little reported so far, were present in HT-29 cells. These isoforms have been, in previous studies, associated with augmented binding affinity of StxB [87] and seemed to be highly relevant for the scission of StxB-induced tubular membrane invaginations [88]. In Ramos leukaemic cells, the non-saturated isoform C24:1 constituted 39% of the total Gb3. The saturated C16:0 and 24:0 isoforms represented around 20% each, the C22:0 isoform only 9% and the C18:0 isoform around 5% of the total Gb3 amount. Small traces of other isoforms included C16:0-OH, C18:1, C20:0, C24:1-OH, C22:0-OH and C24:0-OH (Fig. 9c). In MDA-MB-231 triple-negative breast cancer cells, 42% of the isoforms were designated to C24:0 and 26% to its unsaturated form C24:1. About 22% were matched to the C16:0 and only 7% to the C22:0 Gb3 isoform. The remaining C16:0-OH, C18:1, C20:0, C24:1-OH, C22:0-OH and C24:0-OH were detected only in trace amounts (Fig. 9d). In Namalwa and LS-174 cells, hardly any Gb3 isoform was detected (Fig. S8). These results are alternatively depicted as a heat map in Fig. S8, clearly showing that each model cell line has a unique profile of Gb3 isoforms.

It has been reported that the length and degree of saturation of different Gb3 isoforms affect the binding of StxB and LecA in their native conformation [48, 87, 89, 90]. However, the influence of the structure of each Gb3 isoform on lectin-CAR T cell binding is entirely unknown. Thus, we performed a correlation analysis between the abundance of each Gb3 isoform and the cytotoxicity of different lectin-CAR T cells. We aimed to identify Gb3 isoforms that might be preferentially recognised by a given lectin-CAR. The correlation coefficients are represented as a heat map (Fig. 9e). According to this, the Shiga-CAR T cells seemed to prefer the C18:1, C16:0, and C22:0 isoforms. Hydroxylated Gb3 isoforms reduced recognition by nearly 50%. In contrast, the hydroxylation of the C24:1 isoform increased 3 times the recognition (0.50) by Shiga-CAR T cells compared to the non-hydroxilated (0.17). LecA-CAR T cells correlation values suggest a better recognition of the C24:0, C16:0 and the C18:1. Again, the hydroxylated isoforms were less preferred, reducing the specific killing almost to half. The Mitsuba-CAR T cells equally favoured C18:1, C22:0 and C24:0, and recognition of hydroxylated forms were also reduced. Taken together, we observed that the C18:1, C24:0, C16:0, and C22:0 have the highest correlation coefficients and may be the best-recognised isoforms. Strikingly, the C18:1 is present in very low amounts in the three model cell lines. In summary, the correlation analysis indicates that the specific recognition of Gb3 by lectin-CARs is robust as they could target a wide variety of Gb3 isoforms present in the target cells.

## Discussion

One of the biggest challenges in oncology is to find novel molecular targets and strategies to selectively target and eliminate cancer cells. Therapies targeting tumour-associated carbohydrate antigens in combination with the anti-cancer efficacy of CAR T cells may unlock attractive and powerful treatment options hitherto underutilised. In light of this, we explored a strategy to target the glycosphingolipid Gb3 using novel lectin-based CARs. Our study demonstrates that lectin-CAR T cells, which specifically recognise Gb3, induced the killing of several cancer types. Our approach serves as proof of concept for developing specific lectin-CARs targeting other tumour-associated carbohydrate antigens. Moreover, targeting of TACAs could be relevant in combinatorial therapies, as they target glycans on the cell surface or in the tumour vasculature, thus, facilitating or enhancing the anti-cancer activity of other immunotherapeutics or drugs as it has been carried out with therapies targeting GD2 and MUC1 [91, 92].

We created a collection of lectin-CARs that employed distinct Gb3-binding lectins as binding domains, such as the lectin LecA from *P. aeruginosa*, the B-subunit of Shiga toxin mainly produced by *S. dysenteriae* and *Escherichia coli* strains, and the computationally designed lectin Mitsuba [43, 45]. The successful use of StxB for in vivo targeting of Gb3^+^ xenografts [40, 93–95] and the study of LecA as a drug delivery system [21, 96] in Gb3^+^ cells inspired the design of the lectin-CARs.

Lectin-CAR T cells, in particular those expressing Shiga-CARs, exhibited cytotoxicity against the Burkitt's lymphoma-derived cell lines [Ramos (75.5%), Raji (26%) and Daudi (40%)], which, like other B-lymphoma cell lines, also express and accumulate Gb3 at their cell surfaces [32]. Lectin recognition of the different Gb3 isoforms expressed in a cell determines the killing efficiency of the lectin-CAR T cells. It has been shown that SxtB and LecA have different preferences to recognise specific Gb3 isoforms [75, 76]. Cell lines with poorly recognised Gb3 isoforms are unlikely to be eliminated, which may be the case of the Raji cells.

Interestingly, Shiga-CAR T cells showed that they could reach a cytotoxic activity comparable to anti-CD19-CAR T cells (> 75%). CAR-T cell therapy has achieved good objective responses in diffuse large B cell lymphoma, although their efficacy in adult Burkitt's lymphoma remains limited to a few reports with variable response rates. In these case reports, anti-CD19-CAR T cells have been used to target malignant and healthy B cells, often leaving the patients with long-term B-cell aplasia and hypogammaglobulinemia. Hypogammaglobulinemia is a pathological decrease in antibody production which renders patients susceptible to potentially life-threatening infections [97, 98]. Studies have demonstrated that anti-CD19-CAR T cells could persist for years in patients requiring expensive and recurrent treatments to reconstitute their immunoglobulin levels and protect against opportunistic infections [99, 100]. Lectin-CARs might thus represent an alternative and a potential treatment for haematological malignancies to overcome limitations such as life-threatening-associated toxicities, poor responses to anti-CD19-CAR T cells, antigen escape and resistance in B cell malignancies.

Besides B cell malignancies, the lectin-CAR T cells displayed anti-tumour activity towards the colorectal cancer cell lines HT-29 and HTC-116. A killing efficacy of up to 99.8% was achieved by Shiga-CAR and 92% by Mitsuba-CAR, respectively. Colorectal cancer is the cancer type with the highest number of patients analysed for Gb3 expression. These studies showed an increase in Gb3 expression compared to normal or benign colonic tissue [33, 35, 42]. Also, it has been reported that colon cancer metastasis is Gb3-dependent [42]; therefore, targeting Gb3 can inhibit the progression of these tumours.

We have also investigated the cytotoxicity of lectin-CAR T cells toward the MDA-MB-231 triple-negative breast cancer cells. Triple-negative breast cancer is the most aggressive and lethal breast cancer subtype, for which, to date, we are lacking successful targeted treatments [82, 101]. Lectin-CAR T cells, particularly the LecA-CAR, effectively recognised and lysed MDA-MB-231 cells with a killing efficacy of 59.7%. Most primary breast cancer tumours and breast cancer cell lines (70–80%) overexpress Gb3 compared to normal tissue [38, 102, 103]. Notably, the lectin-CAR T cells were specific for Gb3 and did not exhibit off-target cytotoxicity as demonstrated using Gb3^−^ tumour cells such as LS-174 and JIMT1.

The notable performance of the lectin-CAR T cells against cells derived from solid tumours and haematological malignancies promises a comprehensive application. Gb3 is present in many additional tumour types such as lung, testicular, and ovarian cancer [40, 54]. In addition to directly targeting the tumour cells, lectin CAR-T cells might provide a benefit by targeting the tumour vasculature because Gb3 has been predominantly found in tumour blood vessels [104, 105]. In fact, a neuroblastoma study demonstrated that Gb3 targeting prevented tumour angiogenesis [106]. Gb3 has also been associated with multi-drug resistance in lung and breast cancer and chronic myeloid leukaemias [55, 57, 107]. Gb3 is co-expressed and interplays with efflux transporters such as the ATP-binding cassette, resulting in the active efflux of several chemotherapeutic agents. These anti-cancer agents simultaneously enhance the expression of Gb3 and multi-drug resistance-related proteins, resulting in a severely reduced effectivity of chemotherapeutic drugs [55, 108, 109]. Lectin-CAR T cells could be used as second-line therapy in the treatment of drug-resistant cancers.

There are many scenarios in which the Gb3-binding lectin-CARs could be applied, considering that the number of effective treatments for solid tumours is still very limited. The feasibility of the application of lectin-CAR T cells against cancer depends on at least two Gb3-related factors: the relative abundance of Gb3 on the cell surface of normal and cancer cells and the heterogenicity in the structure of Gb3, which defines its distribution within the cell membrane, the exposure of the carbohydrate head of Gb3, and as a consequence, the degree of recognition by different lectin-CAR T cells. Firstly, our MS analysis revealed that Gb3 abundance varied between model tumour cell lines; Gb3 is at least 30% more abundant in HT-29 cells than in Ramos and MDA-MB-231 cells. In comparison, Gb3 was barely detected in Namalwa and not detected in the LS-174 cells. Our analysis exposed a strong-to-moderate dependency between Gb3 abundance and specific killing by the lectin-CAR T cells, especially when pure populations of lectin-CAR T cells were employed. Thus, Gb3 abundance determines lectin binding, as previously reported [90], thereby the recognition and cytotoxic activity of the lectin-CAR T cells. Secondly, the natural heterogenicity of Gb3 regarding chain length, degree of saturation and hydroxylation of its fatty acyl chain also impact lectin binding [30, 76, 83, 87, 90]. To identify whether the structural variability of Gb3 also influences the ability of the lectin-CAR T cells to target Gb3^+^ cancer cell lines, we performed an MS screening to characterise the Gb3 isoforms in each of the selected cells lines. We then completed a correlation analysis between Gb3 isoforms and the cytotoxicity of lectin-CAR T cells. The MS analysis brought to light the heterogeneity of Gb3 and revealed distinct Gb3 isoform profiles for the different model cell lines. In Ramos and MDA-MB-231 cells, the most abundant Gb3 isoforms were Gb3(d18:1/C16:0), Gb3(d18:1/C24:0) and Gb3(d18:1/C24:1), in line with a previous report [110]. In contrast, the most common isoforms present in the HT-29 cell line were Gb3(d18:1/C16:0), Gb3(d18:1/C24:0) and, unexpectedly, Gb3(d18:1/C16:0-OH). Additional species with little presence, e.g., Gb3(d18:1/C22:0) and Gb3(d18:1/C18:1), were also detected and considered in further analysis. These species, although marginally expressed, may play a critical role in lectin-CAR recognition. On the one hand, the highest correlation coefficient with the cytotoxicity of Shiga-CAR T cells was obtained for Gb3(d18:1/C18:1), Gb3(d18:1/C16:0), and Gb3(d18:1/C22:0), which probably are the preferred Gb3 isoforms. Following these results, it has been reported that C18:1 and C22:0 were the most effective fatty acyl chains for StxB binding, in particular at lower receptor concentrations [87, 90]. According to the calculated correlation coefficients, it seems that the hydroxylated Gb3 species, which were not yet very often described, also play a role in the binding and activation of Shiga-CAR T cells. In line with Schütte et al. and Römer et al., who have shown that hydroxylated species are important for the scission of StxB-induced membrane tubules [88, 111]. On the other hand, the highest correlation coefficient for the LecA-CAR T cells was registered for the Gb3(d18:1/C24:0) isoform. This result is consistent with a recent report, suggesting that Gb3(d18:1/C24:0) is indeed the preferred Gb3 isoform of LecA [48]. Taken together, the Shiga-CAR and LecA-CAR T cells well reflected the Gb3 preferences previously described for StxB and LecA in their native forms. In contrast, the preferences of the engineered Mitsuba lectin for the different Gb3 isoforms have not been studied yet. The highest correlation coefficients for the Mitsuba-CAR T cells were found for Gb3(d18:1/C18:1), Gb3(d18:1/C24:0) and Gb3(d18:1/C22:0). These data may thus give a hint to the preferred species of this lectin for future studies. Interestingly, each of the lectin-CAR T cells studied herein recognised several Gb3 species. This ability could be an advantage since the heterogeneous expression of an antigen is a major limitation for effective tumour targeting by CAR T cells [112].

In our study, the recognition of the Gb3 isoforms might also be influenced by the efficiency of the lectin-CAR constructs to be expressed on the T cell surface. The LecA-CAR was detected at high levels on the cell surface of primary T cells, while the Shiga-CAR and Mitsuba-CAR were detected at lower levels. The low detection could be due to different reasons, e.g., an alternative protein folding preventing the binding of the anti-FLAG antibody; it might also be the consequence of CAR degradation or less efficient export to the plasma membrane due to the lectin domain of bacterial or artificial origin, as well as their molecular weight and native conformation, e.g. Mitsuba. Regardless little is known about the expression of bacterial proteins in mammalian cells, it has been reported that some degree of glycosylation, specifically N-glycosylation, is required for the correct folding of bacterial proteins destined for the extracellular space [113]. We must also consider that human glycosylation patterns can modify the lectin domains. Additionally, the cryptic signals present in bacterial gene sequences might negatively impact the intracellular trafficking pathways of mammalian cells [114]. Altogether, this might explain the lower detection level of Gb3-binding lectin-CARs on the cell surface. A similar phenomenon has been observed for the expression of the cholera toxin B-subunit and the tetanic toxin C in mammalian cells [115, 116]. Despite that, the expression levels were enough to perform efficient CAR T cell recognition, activation and elimination of the target cells.

The use of lectins as a binding motif in CAR T cells can be complementary or alternative to other therapeutic approaches such as antibodies targeting TACAs. On one side, generating antibodies with a high affinity for TACAs can be challenging due to the low immunogenicity and immune tolerance of these structures. On the other side, lectins naturally and specifically recognise TACAs [16, 117, 118]. The engineering of lectins opens up a world of possibilities with lectins of tailored valency and affinity [119], a different number of subunits [120] or reduced immunogenicity, e.g., Mitsuba [46], that would allow broader use of these carbohydrate-binding proteins.

## Conclusions and outlook

In summary, we demonstrate that Gb3-binding lectin-CAR T cells recognise multiple Gb3 isoforms present in different cancer types. The cytotoxicity of Shiga-CAR T cells against several Burkitt's lymphoma and solid tumour cells was the most prominent among the panel of studied lectin-CARs. However, LecA-CAR and Mitsuba-CAR T cells also showed promising activity in specific cases, possibly due to a delimited recognition of Gb3 species.

In vivo studies will be conducted in the near future to investigate the behaviour of Gb3-binding lectin-CAR T cells in model organisms. They would also help to clarify open questions regarding which specific lectin to best use, and which Gb3 isoform is safest and most effective for targeting. In addition, unanswered questions regarding the off-targeting toxicities of the lectin-CAR T cells could be addressed. Although tumour imaging studies in mice with StxB did not show extensive off-targeting [93–95, 121], still there is a reasonable risk of targeting normal tissue in which Gb3 is also expressed, e.g. kidney [122], nervous system [123], liver or intestine [53, 124]. The clinical translation of these findings remains to be done; our study is designed as a proof-of-concept, and it has herewith demonstrated the feasibility of targeting cancer cells by using novel and innovative lectin-CAR constructs. Other TACAs and lectins should be studied, pointing to tailor-made lectin tools with improved affinity, specificity and immunogenicity [46, 125, 126]. Lectin engineering is a great opportunity to discover and develop innovative therapeutic tools.

## Supplementary Information

Below is the link to the electronic supplementary material.

Supplementary file1 (DOCX 6023 KB)

Supplementary file2 (DOCX 14 KB)

Supplementary file3 (MP4 245766 kb)

Supplementary file4 (MP4 251910 kb)

## Data Availability

The datasets generated and/or analysed during the current study are available from the corresponding authors on reasonable request.

## Abbreviations

BLI: Bioluminescence
CAR: Chimeric antigen receptors
DMEM: Dulbecco's Modified Eagle Medium
DOPC: 1,2-Dioleoyl-sn-glycero-3-phosphocholine
DOPE: 1,2-Dioleoyl-sn-glycero-3-phosphoethanolamine
ELISA: Enzyme-linked immunoabsorbent assay
E:T: Effector to target
PBS: Phosphate-buffered saline
Gb3: Globotriaosylceramide
GSL: Glycosphingolipid
GUV: Giant unilamellar vesicles
HBSS: Hanks' balanced salt solution
IFN-γ: Interferon γ
IMDM: Iscove's Modified Dulbecco's Medium
IS: Internal standard
ITO: Indium tin oxid
LC: Liquid chromatography
MFI: Mean fluorescence intensity
MOI: Multiplicity of infection
MS: Mass spectrometry
NT: Non-transduced cells
NEAA: Non-essential amino acids
PBMC: Peripheral blood mononuclear cell
PPMP: 1-Phenyl-2-palmitoylamino-3-morpholino-1-propanol
RLU: Relative light units
RPMI: Roswell Park Memorial Institute
scFv: Single-chain variable fragment
SDS: Sodium dodecyl sulfate
StxB: Shiga toxin B-subunit
TACAs: Tumour-associated carbohydrate antigens
TNF-α: Tumour necrosis factor α
