# Supplementary material for: Novel lectin-based chimeric antigen receptors target Gb3-positive tumour cells
Source: Cell Mol Life Sci. 2022 Sep 12;79(10):513. doi: 10.1007/s00018-022-04524-7 (PMC9468074; doi:10.1007/s00018-022-04524-7)
Supplement: Supplementary file 1 — Supplementary file1 (DOCX 6023 KB) [file 18_2022_4524_MOESM1_ESM.docx]

Supplementary Figures

**Novel lectin-based chimeric antigen receptors target Gb3-positive tumour cells**

Ana Valeria Meléndez^1,2,3,4^, [Rubí M-H Velasco Cárdenas](https://pubmed.ncbi.nlm.nih.gov/?term=C%C3%A1rdenas+RMV&cauthor_id=32690949)^1,2,3^, Simon Lagies^5^, Juliane Strietz^6^, **Lina Siukstaite^1,2,3^, Oliver S. Thomas^1,2,3,4^, Jana Tomisch^1,2,3^, Wilfried Weber^1,2,3,4^, Bernd Kammerer^2,5,7^, Winfried Römer^1,2,3,4,8,*^ and Susana Minguet^1,2,3,4,8,9*^**

^1^ Faculty of Biology, University of Freiburg, Schänzlestraße 1, 79104 Freiburg, Germany

^2^ BIOSS, Centre for Biological Signalling Studies, University of Freiburg, Schänzlestraße 18, 79104 Freiburg, Germany

^3^ CIBSS, Centre for Integrative Biological Signalling Studies, University of Freiburg, Schänzlestraße 18, 79104 Freiburg, Germany

^4^ Spemann Graduate School of Biology and Medicine (SGBM), University of Freiburg, Albertstraße 19a, 79104 Freiburg, Germany

^5^ Institute of Organic Chemistry, Albert-Ludwigs-University Freiburg, Albertstraße 21, 79102 Freiburg, Germany

^6^ CYTENA GmbH, Zollhallenstr. 5, 79106 Freiburg, Germany

^7^ Centre for Integrative Signalling Analysis, University of Freiburg, Habsburgerstraße 49, 79104 Freiburg, Germany

^8^ Freiburg Institute for Advanced Studies (FRIAS), University of Freiburg, Freiburg, Germany

^9^ Center of Chronic Immunodeficiency (CCI), University Clinics and Medical Faculty, Freiburg, Germany

*****corresponding authors

**To whom correspondence should be addressed**:

**Prof. Dr. Winfried Römer,** winfried.roemer@bioss.uni-freiburg.de

PD Dr. **Susana Minguet,** susana.minguet@biologie.uni-freiburg.de


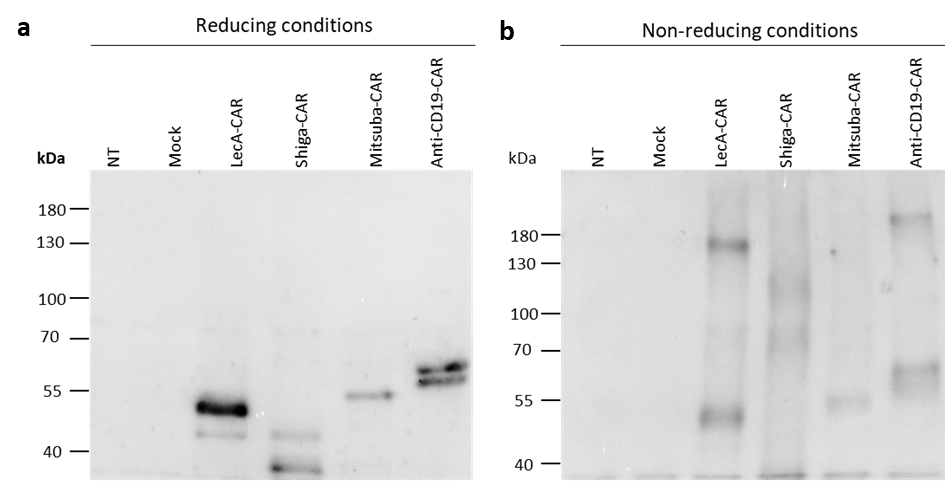


**Fig. S1** SDS-PAGE and immunoblotting of the lectins-CARs and controls**.** Incorporation of the selected CARs in PBMCs. For the analysis, non-transduced (NT) and transduced (with empty vector) mock cells were included as controls, in addition to the panel of the lectin-CARs: LecA-CAR, Shiga-CAR, Mitsuba-CAR and anti-CD19-CAR. Primary T cells were lysed either with reducing SDS buffer (**a**)or non-reducing buffer (**b**)plus 5 μl of urea. Under reducing conditions, all CARs exhibited the expected sizes. Meanwhile, higher molecular weight complexes were observed in non-reducing gels. Despite the presence of identical cysteines in the hinge and transmembrane regions of all CARs, each CAR construct displayed a distinct pattern of oligomerization.

**
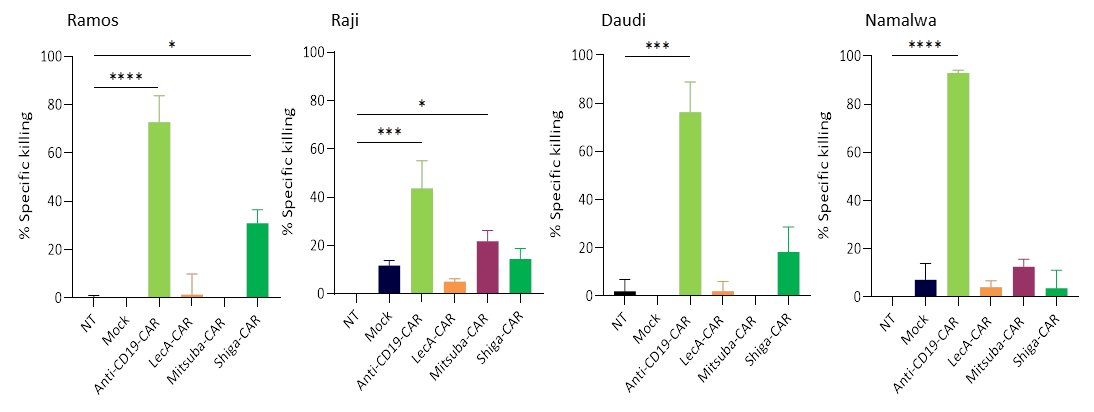
**

**Fig. S2** *In vitro* tumour cell killing by CAR T cells. Burkitt's lymphoma-derived cells lines. After 17 h, the anti-CD19 CAR showed the most prominent cytotoxic activity in all cell lines. The Shiga-CAR specific killing increased and showed 30% for Ramos cells, 14.4% for Raji cells and 18% for Daudi cells. n=3. One-way ANOVA followed by Dunnett's multiple comparisons test. Mean values ± SEM. are shown. **p*  < 0.05, ***p* < 0.01, ****p* < 0.001, *****p* < 0.0001. Data represent at least three independent donors.


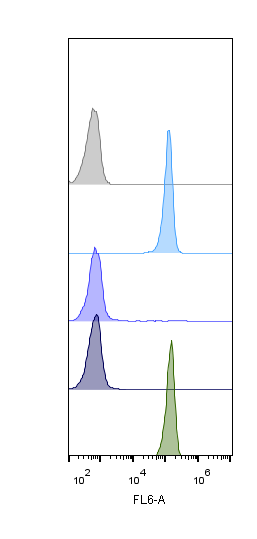


Control

Untreated

PPMP 2 µM

PPMP 1 µM

Vehicle

Gb3

**a**

**b**

**c**

**d**

**Fig. S3** Lectin-CARs specifically recognize Gb3. **a** Gb3 surface expression assayed by flow cytometry in Ramos cells treated with PPMP as indicated. Gb3 was detected by staining with labeled StxB at day 3. Data represent one experiment out of three independent experiments. **b** Ramos Gb3⁺ cells were treated with PPMP for three days and then co-incubated for 24 h with the indicated effector cells: mock, Shiga-CAR and anti-CD19-CAR T cells. Two-way ANOVA followed by Dunnett's multiple comparisons test. Mean values ± s.e.m. are shown. n=3, simultaneously performed co-incubation. **p* < 0.05, ***p* < 0.01 ****p* < 0.001, *****p* < 0.0001, ns, not significant. Representative data of one out of two independent donors **c** CAR expression on the cell surface of T cells was assayed by FLAG-tag staining and flow cytometry analysis upon PPMP treatment for 24 h. Mean values ± s.e.m. are shown. Representative data of one out of two independent donors. **d** PPMP cytotoxicity was assessed using a viability assay in Ramos cells at day 3. Mean values ±  ± SEM are shown. One-way ANOVA followed by Dunnett's multiple comparisons test. **p <*0.05, ***p*< 0.01 ****p*< 0.001, *****p* < 0.0001. Data represent at least three independent experiments.

**Fig. S4** Gb3 expression of the non-tumour Madin-Darby canine kidney (MDCK) and cytotoxicity assay. **a-b** Gb3 expression in MDCK cells, transfected with Gb3 synthase (α1,4-galactosyltransferase), MDCK Gb3⁺ (a), or not MDCK WT (b) was assayed by flow cytometry using fluorescenlty labeled StxB. **c-d** Kinetics of the killing by Shiga-CAR T cells. A cytotoxicity assay was performed using MDCK Gb3⁺ (c) cells and MDCK WT (d) with a E:T ratio 5:1. The Shiga-CAR T cells showed a specific killing of 54% at 17 h. The specific killing towards the MDCK WT cells, not expressing Gb3 synthase, was very low during the assay. These results further support the specificity of the Shiga-CAR T cells towards Gb3⁺cells. Mean values  ± SEM are shown, data of three co-incubations per condition are shown, n=3. Data represent at least two independent experiments


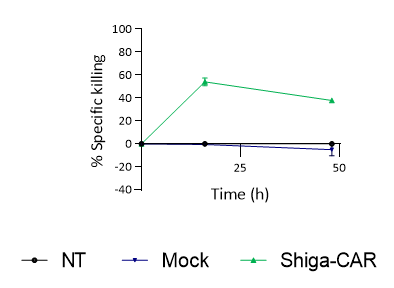

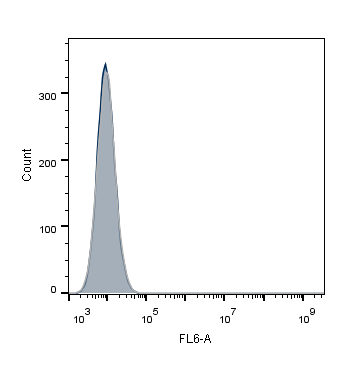

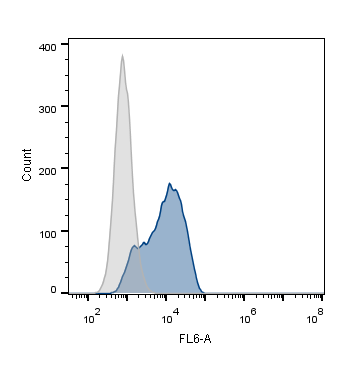


MDCK Gb3⁺

MDCK WT

Gb3

Gb3

**a**

**b**

MFI:16805

62.0%

MFI: 75595

0.0%


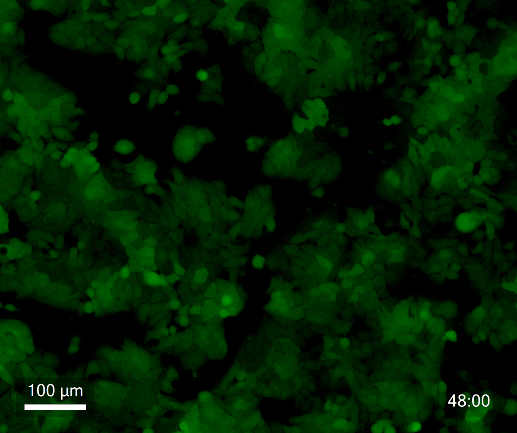

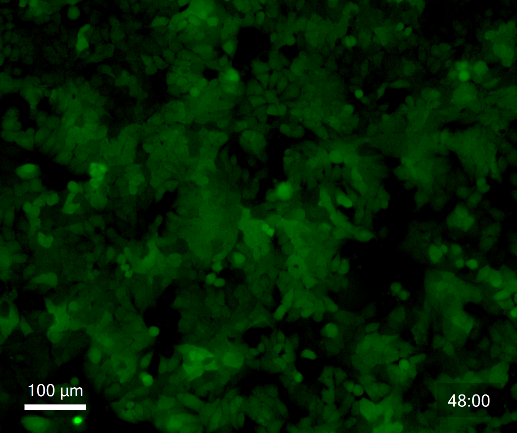

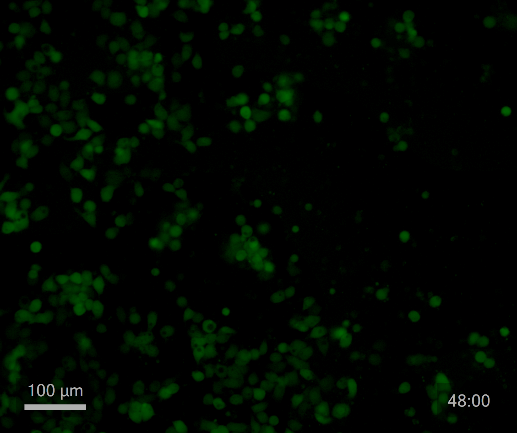


GFP

Mock

Shiga-CAR

NT

C.LIVE Tox Red


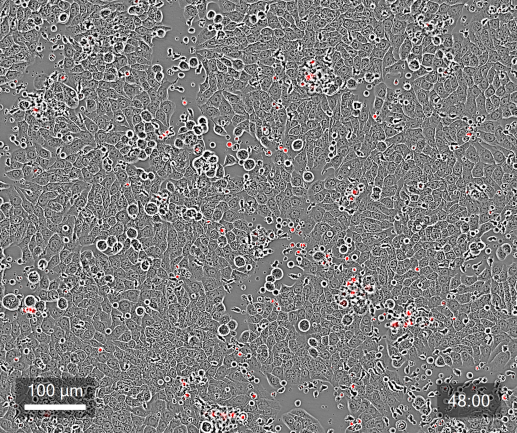

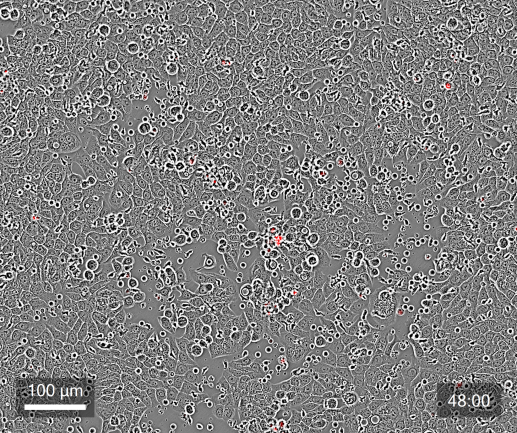

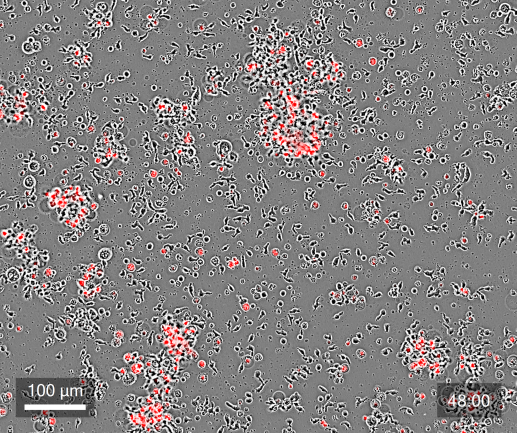


Mock

Shiga-CAR

NT

**a**

**b**

**Fig. S5** Live cell imaging of the Gb3⁺ HT-29 cells in **a** GFP⁺and **b** C.LIVE Tox Red stained cells co-incubated with NT, Mock or Shiga-CAR T cells. Representative images of 48 h are depicted. Scale bar: 100 µm.


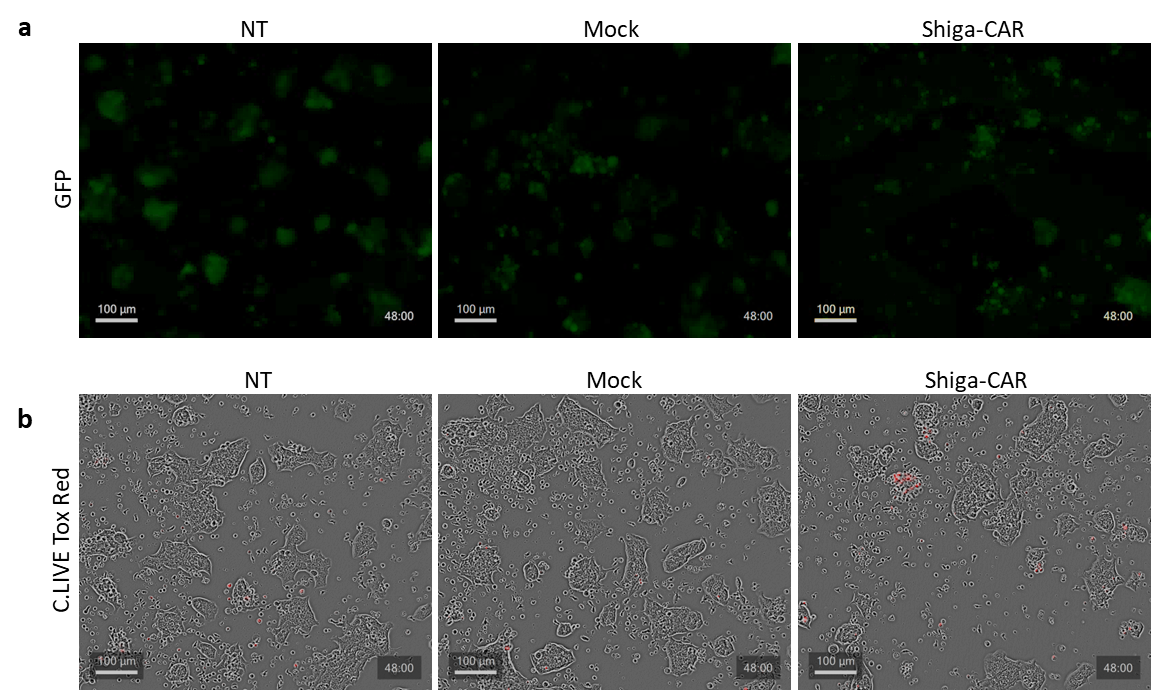


**Fig. S6** Live cell imaging of the Gb3⁻ LS-174 cells in **a** GFP⁺and **b** C.LIVE Tox Red stained cells co-incubated with NT, Mock or Shiga-CAR T cells. Representative images of 48 h are depicted. Scale bar: 100 µm.


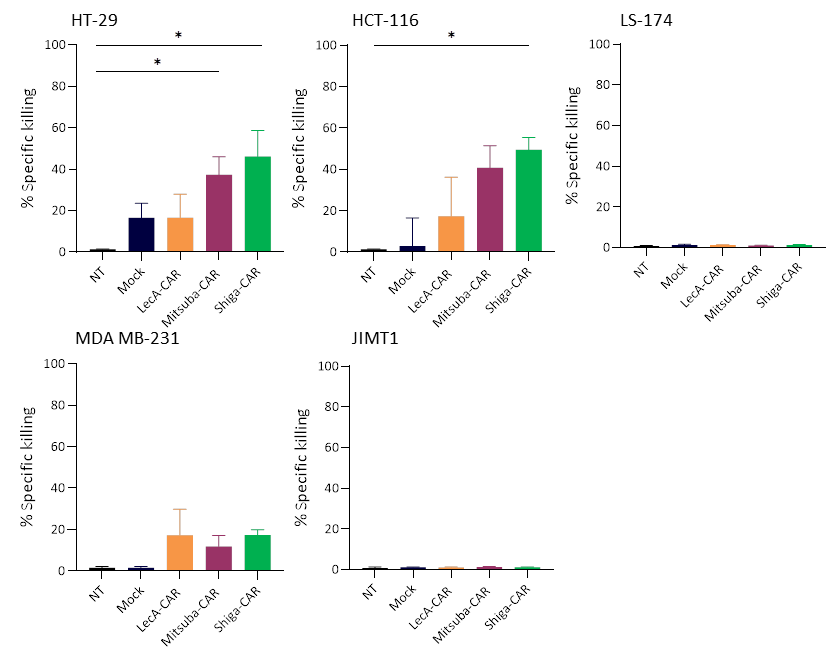


**Fig. S7** *In vitro* solid tumour cell killing by CAR T cells. Specific killing by lectin-CAR T cells of colorectal cancer cell lines (HT-29, HTC-116, LS-174) and breast cancer cell lines (MDA-MB-231 and JIMT1) was checked by bioluminescence assay. After 17 h, the Shiga-CAR and the Mitsuba-CAR appeared to be the most efficient in killing of colorectal cancer cells. One-way ANOVA followed by Dunnett's multiple comparisons test. Mean values ±SEM are shown. **p* < 0.05, ***p* < 0.01, ****p*< 0.001, *****p* < 0.0001. Data represent at least three independent donors.


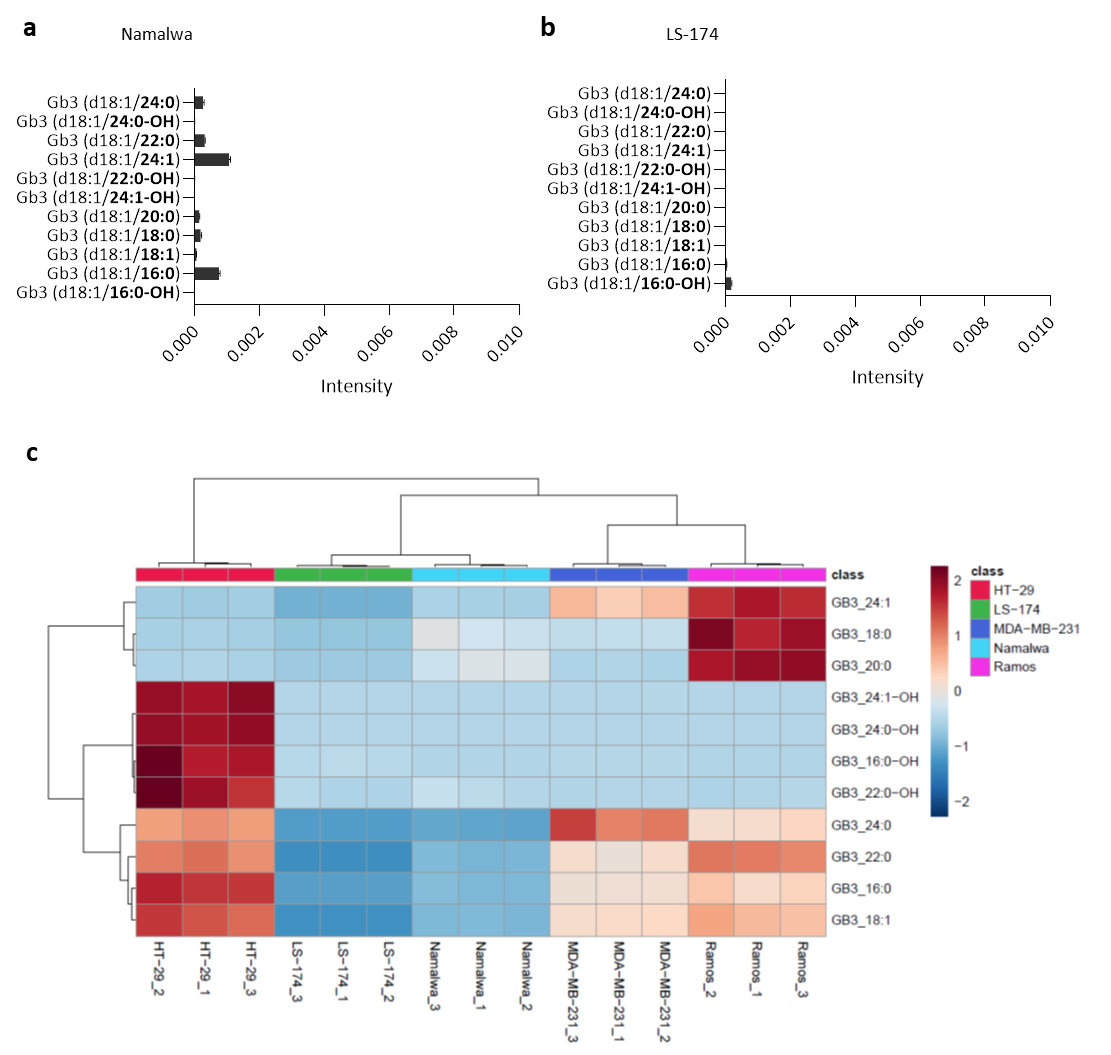


**Fig. S8** Lipid analysis by MS of model cell lines. The analysis revealed the total Gb3 abundance and identified the predominant isoforms for each cell line. a-b Gb3 isoforms present in Namalwa (a) and LS-174 cells (b). Mean values ± SEM are shown. n=3, analyzed samples in one LC-MS sequence. c Heat map of Gb3 content normalized to the internal standard (IS) and the sum of all measured GSL. Range-scaled z scores are displayed.

**
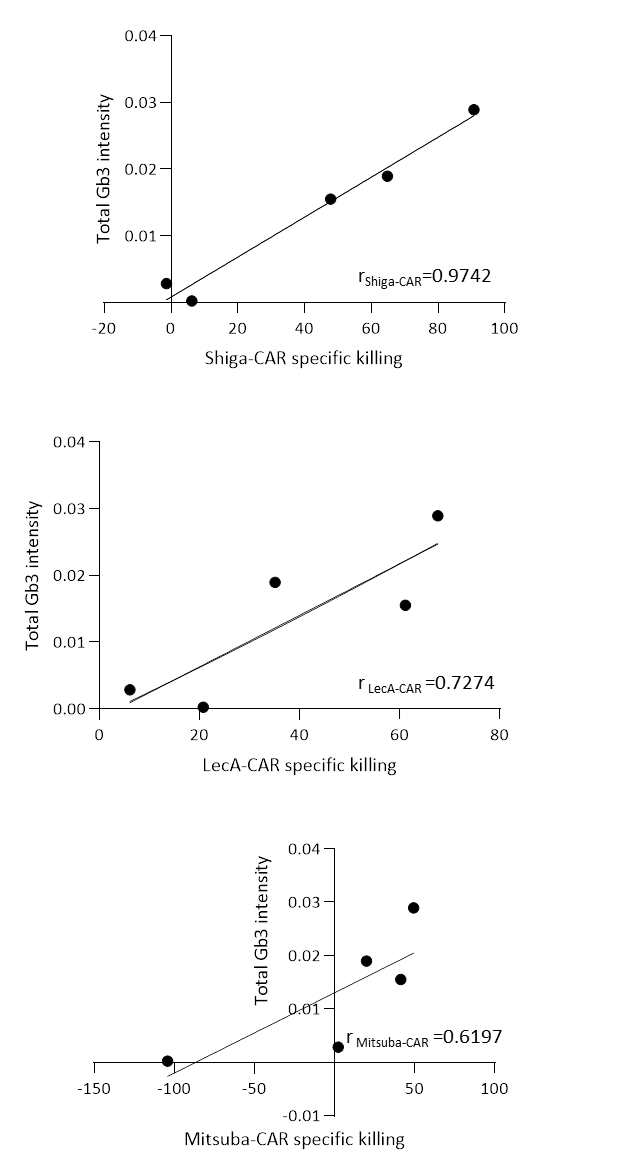
**

**Fig. S9** Pearson correlation between the total Gb3 content of each cell line and the specific killing of the different lectin-CAR T cells. The total Gb3 content considers the sum of the intensities of all Gb3 isoforms detected on each cell line (HT-29, Ramos, MDA-MB-231, Namalwa and LS-174) by MS analysis. The correlation analysis revealed a strong Gb3 dependency for the Shiga-CAR T cell-induced cytotoxicity and moderate dependency for LecA-CAR T cell and Mitsuba-CAR T cells. The higher is the abundance of Gb3 on the target cells; the higher is the cytotoxicity.
