# Supplementary material for: Novel lectin-based chimeric antigen receptors target Gb3-positive tumour cells
Source: Cell Mol Life Sci. 2022 Sep 12;79(10):513. doi: 10.1007/s00018-022-04524-7 (PMC9468074; doi:10.1007/s00018-022-04524-7)
Supplement: Supplementary file 2 — Supplementary file2 (DOCX 14 KB) [file 18_2022_4524_MOESM2_ESM.docx]

Online Resources

**Novel lectin-based chimeric antigen receptors target Gb3-positive tumour cells**

Ana Valeria Meléndez^1,2,3,4^, Rubí M-H Velasco Cárdenas^1,2,3^, Simon Lagies^5^, Juliane Strietz^6^, **Lina Siukstaite^1,2,3^, Oliver S. Thomas^1,2,3,4^, Jana Tomisch^1,2,3^, Wilfried Weber^1,2,3,4^, Bernd Kammerer^2,5,7^, Winfried Römer^1,2,3,4,8,*^ and Susana Minguet^1,2,3,4,8,9*^**

^1^ Faculty of Biology, University of Freiburg, Schänzlestraße 1, 79104 Freiburg, Germany

^2^ BIOSS, Centre for Biological Signalling Studies, University of Freiburg, Schänzlestraße 18, 79104 Freiburg, Germany

^3^ CIBSS, Centre for Integrative Biological Signalling Studies, University of Freiburg, Schänzlestraße 18, 79104 Freiburg, Germany

^4^ Spemann Graduate School of Biology and Medicine (SGBM), University of Freiburg, Albertstraße 19a, 79104 Freiburg, Germany

^5^ Institute of Organic Chemistry, Albert-Ludwigs-University Freiburg, Albertstraße 21, 79102 Freiburg, Germany

^6^ CYTENA GmbH, Zollhallenstr. 5, 79106 Freiburg, Germany

^7^ Centre for Integrative Signalling Analysis, University of Freiburg, Habsburgerstraße 49, 79104 Freiburg, Germany

^8^ Freiburg Institute for Advanced Studies (FRIAS), University of Freiburg, Freiburg, Germany

^9^ Center of Chronic Immunodeficiency (CCI), University Clinics and Medical Faculty, Freiburg, Germany

*****corresponding authors

**To whom correspondence should be addressed**:

**Prof. Dr. Winfried Römer,** winfried.roemer@bioss.uni-freiburg.de

PD Dr. **Susana Minguet,** susana.minguet@biologie.uni-freiburg.de

**Online Resource 1** Interaction of Gb3-GUVs and mock T cells in live-cell imaging experiments. GUVs and mock T cells were co-incubated for 30 minutes. Mock T cells surrounded and approached GUVs over time. Time series were analysed to calculate the contact percentage between GUVs and mock cells. Scale bar: 10 μm. The time of co-incubation is indicated in minutes.

**Online Resource 2** Interaction of Gb3-GUVs and Shiga-CAR T cells in live-cell imaging experiments. GUVs and Shiga CAR-T cells were co-incubated for 30 minutes. Shiga-CAR T cells approached GUVs trying to build contacts. Deformation of GUVs is visible in some areas, which indicates tight Shiga toxin and its receptor Gb3 interaction. Time series were analysed to calculate the contact percentage between GUVsand mock cells. Scale bar: 10 μm. The time of co-incubation is indicated in minutes.
